# *Tenacibaculum maritimum* can boost inflammation in *Dicentrarchus labrax* upon peritoneal injection but cannot trigger tenacibaculosis disease

**DOI:** 10.3389/fimmu.2024.1478241

**Published:** 2024-10-14

**Authors:** Inês A. Ferreira, Paulo Santos, Javier Sanz Moxó, Carla Teixeira, Ana do Vale, Benjamin Costas

**Affiliations:** ^1^ Interdisciplinary Centre of Marine and Environmental Research (CIIMAR), University of Porto, Porto, Portugal; ^2^ Abel Salazar Institute of Biomedical Sciences (ICBAS), University of Porto, Porto, Portugal; ^3^ Fish Immunology and Vaccinology Group, i3S- Instituto de Investigação e Inovação em Saúde, Universidade do Porto, Porto, Portugal

**Keywords:** aquaculture, tenacibaculosis, infection route, innate immunity, gene expression

## Abstract

**Introduction:**

Despite being a bacterial pathogen with devastating consequences, *Tenacibaculum maritimum*’s pathogenesis is not fully understood. The aim of the present study was to elucidate if different inoculation routes (intraperitoneal - i.p - injection and bath challenge - known to induce mortality) can induce tenacibaculosis (i.e., using the same *T. maritimum* inoculum), as well as evaluate the short-term immune response of European sea bass (*D. labrax*). Additionally, the host response against i.p. injection of extracellular products (ECPs) was also studied.

**Methods:**

Fish were i.p. challenged with 5.5 × 10^5^ CFU mL^-1^ of *T. maritimum* cells with or without ECPs (BECPs and BWO, respectively), ECPs alone or marine broth (mock). Another group of fish was bath-challenged with 5.5 × 10^5^ CFU mL^-1^ to confirm the virulence of the bacterial inoculum. Undisturbed specimens were used as controls. The severity of both challenges was determined by following percentage survival. Blood, liver and head-kidney samples were collected at 0, 3, 6, 24 and 48 h post-challenge for assessing immune parameters, oxidative stress and gene expression. Total and differential peritoneal cell counts were performed. The presence of viable bacteria in the blood and peritoneal cavity was studied.

**Results:**

Symptoms of tenacibaculosis, such as skin/fin abrasions, were only observed in the bath-challenged fish, where 0% survival was recorded, whereas 100% survival was observed after i.p. injection of the same bacterial inoculum. An increase in total leukocyte numbers in the peritoneal cavity was observed 3 h post-injection of BECPs when compared to the other treatments. Blood total leukocytes, lymphocytes, and thrombocyte numbers dropped after the challenge, mainly in fish challenged with BECPs. At 48 h post-challenge, bactericidal activity in the plasma increased in fish injected with bacteria (with and without ECPs). The same tendency was seen for some of the oxidative stress parameters.

**Discussion/Conclusions:**

The increased expression of *il1β*, *il6, il8, and hamp1* in fish challenged with ECPs and BECPs suggests a more exacerbated pro-inflammatory response in the head-kidney against these inocula. The infection trial and the observed immune responses showed that the infection route is a determinant factor regarding *T. maritimum*-induced pathogenesis in European sea bass.

## Highlights

- Intraperitoneal injection of *T. maritimum* plus ECPs induced a significant and fast increase in peritoneal cells’ numbers, whereas no changes occurred after injection of ECPs.
- Intraperitoneal injection of *T. maritimum* plus ECPs or ECPs led to increased expression of the inflammatory mediators *il1β*, *il6*, *il8*, *tnfα* and *hamp1* in the head-kidney.
- The infection route is a determinant factor for *T. maritimum* pathogenesis development.

## 1 Introduction

Bacterial diseases are one of the significant constraints to the global aquaculture industry (1). In aquaculture sites, the prevalence of bacteria is high (2), and disease monitoring can become rather complex due to the ability of opportunistic pathogens to asymptomatically colonize farmed species as an integral component of “healthy” microbiome (3, 4).

*Tenacibaculum maritimum* (Family Flavobacteriaceae, Phylum Bacteroidetes) is pathogen that threatens the production of many economically important fish species, such as European seabass (*D. labrax*) (5), Gilthead seabream (*Sparus aurata*) (6), turbot (*Scophthalmus maximus*), Senegalese sole (*Solea senegalensis*) (7), and Atlantic salmon (*Salmo salar*) (8). It induces small lesions, upraised spots, scale loss and some disintegration of the epidermis in the fish body surface, namely in the head, skin or fins (9–12). The extensive skin lesions, together with the gill abrasions, offer a matchless chance for other opportunistic pathogens to enter the host, leading to secondary infections, some of which can culminate in systemic infections (11, 13). Due to mortality rates and tenacibaculosis symptomatology, the global economic losses associated with this pathology are considerable (11, 14).

Although several studies have been carried out to understand better *T. maritimum*’s pathogenicity, its transmission, route of infection and the dynamics established between the pathogen and host are not fully disclosed. Over the last few years, different experimental infection methods and inocula resulted in distinctive rates of mortality (11, 15–18). Among the different types of inocula, several studies have used *T. maritimum* extracellular products (ECPs) to ascertain their potential immunogenic effects (13, 19, 20). The ECPs have been described as a virulence mechanisms used by *T. maritimum* to facilitate erosion, colonization and invasion of the host’s tissues (11, 13, 21). Indeed, ECPs contain toxins with high proteolytic and cytotoxic activities *in vitro* and high toxicity *in vivo* (13, 20, 22). Escribano et al. (23) performed *in vitro* trials using an epithelioma papulosum cyprini (EPC) cell line to define the cytotoxicity of different doses of ECPs extracts: total ECPs, OMVs, and soluble ECPs (at the same protein concentration, 0.5 mg ml^-1^). All extracts displayed dose-dependent cytotoxic effects. However, the cytotoxicity effect was higher for total ECPs than for OMV or S-ECP fractions (23). Moreover, qualitative effects in sole fingerlings subcutaneously injected with total ECPs, OMVs, or S-ECPs confirmed the higher toxicity of total ECPs, which induced ulcerative and hemorrhagic lesions between 12 and 24 h after the challenge (23). Although these studies support the active role of ECPs in *T. maritimum* virulence, their effects on the host have not been fully explored.

The first experimental studies of tenacibaculosis were conducted in the 1990s, and they focused on commercial fish species and different infection methods. Initially, abrasion/scarification of fish skin, followed by smearing these lesions with pure broth culture, was used to experimentally induce tenacibaculosis (24). Later on, Powel et al. (18) used the abrasion method to directly inoculate high concentrations of *T. maritimum* (4×10^11^ cells *per* fish) on the gills of Atlantic salmon smolts to induce and enhance necrotic branchitis of the gill epithelium successfully. Other methods, such as subcutaneous injection (25), cohabitation (15), and prolonged immersion (16, 26) were also tested, leading to different degrees of mortality and symptomatology. More recently, Faílde et al. (27) experimentally infected turbot (*Scophthalmus maximus*) using the subcutaneous and intraperitoneal (i.p.) routes, demonstrating that they are both able to cause bacteremia in fish. For the group subcutaneously challenged with *T. maritimum*, extensive areas of necrosis were observed in the muscles, with an inflammatory response in the inoculation site; degeneration of muscle fibers was also detected with scattered inflammatory cells in these necrotic areas (27). For the i.p. challenged group, no lesions were observed in the skin or muscle throughout the study; however, the organs in the coelomic cavity exhibited inflammatory response and necrosis in the spleen, kidney, liver, and gastrointestinal tract (27). Finally, Avendaño-Herrera et al. (13) reported a sole isolate that could not induce mortality in turbot fry challenged by both immersion and i.p. routes. This inability to consistently induce tenacibaculosis under experimental conditions underscores the importance of disclosing the factors behind its pathogenesis, disease development, and host immune response. The aim of the present study was two-fold. On the one hand, it was intended to elucidate if different inoculation routes (intraperitoneal injection and bath challenge - known to induce mortality) can induce tenacibaculosis (i.e. using the same inoculum), whereas on the other hand, it also aimed to evaluate the short-term innate immune response of European sea bass (*D. labrax*) when challenged through these two different challenge models. As a further step, the host response against i.p. injection of ECPs was also studied.

## 2 Materials and methods

### 2.1 Bacterial culture and inoculum preparation

This study used a *T. maritimum* strain (ACC13.1, serotype O3) that was previously isolated from Senegalese sole (*Solea senegalensis*) during a farm outbreak (28). Preceding studies with this strain involving bath challenge as an infection model confirmed the pathogenicity of this isolate (16, 29). The strain was supplied by Professor Alicia E. Toranzo (Departamento de Microbiología y Parasitología, Facultad de Biología, University of Santiago de Compostela, Spain). Recovery from the frozen stocks at −80°C was done using marine agar (MA; Laboratories CONDA, Spain) at 25°C for 48 h.

For preparing the inoculum, 50 mL of marine broth (MB; Laboratories CONDA, Spain) was inoculated with bacteria in a 500 mL Erlenmeyer, grown at 25°C, with continuous shaking (180 rpm) for 48 h. Turbidity was measured at 600 nm (Spectrophotometer, UV-1600PC, VWR), and exponentially growing bacteria (OD = 0.613) were collected. For the bath challenge, bacteria were collected by centrifugation at 3,000 *× g* for 10 min, resuspended in MB, and adjusted to a 5.5 *×* 10^5^ CFU mL^-1^. For the i.p. challenge using whole cells without *T. maritimum*’s ECP, bacteria were centrifuged at 3,000 *× g* for 10 min, the obtained pellet was resuspended in MB and adjusted to a 5.5 *×* 10^6^ CFU mL^-1^ (treatment designated by BWO). For the i.p. challenge using cells with *T. maritimum*’s plus ECP, bacteria were adjusted to 5.5 *×* 10^6^ CFU mL^-1^ without any washing procedure (treatment designated by BECPs). The bacterial concentration was adjusted with a predetermined growth curve for this strain: y = 2 × 10^8^x + 4 × 10^7^, where the x corresponds to turbidity at 600 nm (OD) and y to the bacterial concentration (CFU mL^-1^).

### 2.2 Preparation of extracellular products

To obtain *T. maritimum*’s ECPs, bacteria were cultured as previously described until OD600 = 0.646 (exponential phase) and centrifuged at 4,000 *× g* for 30 min at 4°C. Culture supernatant was filtered using a 0.2 µm pore-size Vacuum Filtration System (VWR, USA), concentrated approximately 20-fold using Amicon ultra-15 centrifugal filter units (10 KDa cut-off) (Merck Millipore, Germany) according to the manufacturer´s instructions, aliquoted and stored at -80°C. The protein concentration concentrated ECPs was determined using the Pierce™ BCA Protein Assay kit (Thermo Fischer Scientific USA), with bovine serum albumin as standard. The protein profile of the ECPs was analyzed by SDS-PAGE after trichloroacetic acid (TCA) precipitation. Shortly, proteins from 1 mL aliquots of concentrated ECPs were precipitated with 10% (w/v) TCA for 30 min on ice and recovered by centrifugation (19,800 *× g*, 15 min, 4°C). The obtained pellets were washed with 10% (w/v) TCA, centrifuged, washed once more with acetone, allowed to dry, and solubilized in a gel loading buffer (50 mM Tris-HCl pH 8.8, 2% SDS, 0.05% bromophenol blue, 10% glycerol, 2 mM EDTA, and 100 mM DTT), at 95 °C for 5 min. Samples were electrophoresed in a 14% polyacrylamide gel using the Laemmli discontinuous buffer system (30), followed by staining with Coomassie Brilliant Blue (0.2% Coomassie R-250, 50% methanol, 10% acetic acid).

### 2.3 Fish farming and experimental design

The experiments were approved by the CIIMAR Animal Welfare Committee and DGAV (ORBEA-CIIMAR_26_2018) and were carried out under license number 0421/000/000/2020 in a registered facility (N16091.UDER). The current study was conducted under the supervision of researchers accredited in laboratory animal science by the Portuguese Veterinary Authority following FELASA category C recommendations and in agreement with the guidelines on the protection of animals used for scientific purposes according to the European Union directive (2010/63/EU).

European sea bass juveniles (35.6 ± 6.5 g) were obtained from a commercial fish farm (Valencia, Spain) with no record of previous tenacibaculosis outbreaks and were maintained in quarantine for 4 weeks at CIIMAR fish-holding facilities in a recirculated aerated seawater (salinity 32.0 ± 1.8 ‰) system, with 8.6 ± 0.1 mg mL^-1^ dissolved oxygen, and a 12 h light/12 h dark photoperiod. Mechanical and biological filtration was used to maintain the water’s quality, and fish were given a commercial diet (Aquasoja, Portugal) consisting of 2% of their body weight divided into two meals *per* day. Ammonia and nitrite levels were measured daily using commercial kits and kept at 0.7 ± 0.2 and 2.2 ± 1.0 mg L^-1^, respectively. The water temperature was maintained at 20.4 ± 0.2°C until the beginning of the bacterial challenge. At the challenge, the temperature was increased to 25°C to simulate water temperature conditions at which tenacibaculosis outbreaks occur (11).

Before the bacterial challenge, fish were randomly distributed into closed recirculating seawater systems (7.4 kg m^-3^ stocking density), one for the mock-challenged fish and another for the challenged fish, each with three aquaria for sampling purposes (three replicates *per* treatment) and one aquarium *per* treatment to follow percentage survival. An additional system was used for the bath challenge using *T. maritimum* or MB (two replicates *per* treatment) to follow mortality after the bath challenge. After transfer to the experimental aquaria, fish were acclimated for another 4 weeks under the conditions specified above.

Fish were challenged through i.p. injection with 100 µL of MB containing 5.5 *×* 10^5^ CFU *T. maritimum* with or without ECPs (BWO and BECPs, respectively) or with 100 µL of concentrated ECPs (150 µg of protein fish^-1^). The mock-challenged fish were i.p. injected with 100 µL sterile MB. For the bath challenge, fish at a stocking density of 18 kg m^-3^ were immersed for 2 h with vigorous aeration in MB containing 5.5 *×* 10^5^ CFU mL^-1^ of *T. maritimum*. Mortality was followed for 10 days, and dead or moribund animals were collected or euthanized (0.7 mL L^-1^ 2-phenoxyethanol (Merck, ref. 807291, Germany), and counted as dead.

### 2.4 Sampling

After euthanizing the fish with 0.7 mL L^-1^ 2-phenoxyethanol, post-mortem samples were taken (Merck, ref. 807291, Germany). Fish were sampled before starting the i.p. challenge (time 0, control) and at 3, 6, 24 and 48 h post-challenge. At each time point, four fish were sampled from each triplicated tank (n = 12 *per* treatment), and blood was aseptically collected from the caudal vein with heparinized sterile 1 mL syringes. A volume of 10 µL of the collected blood was plated in MA, followed by incubation for 72 h at 25°C to detect the presence of viable *T. maritimum*. The remaining blood was transferred to heparinized 1.5 mL tubes, and one portion was used for hematological analysis, while the remaining blood was centrifuged for 10 min at 10,000 *× g* at 4°C for collecting plasma that was stored at -80°C. Fragments of head-kidney were collected and stored in RNA later (1/10, w/v) at 4°C for the first 24 h and then at -80°C for molecular biology analysis. Liver samples were also collected for oxidative stress analysis and were directly placed in liquid nitrogen and stored at -80°C.

### 2.5 Haematological parameters

According to MaChado et al. (31), the hematological profile was carried out. Total white (WBC) and red (RBC) blood cells were counted using a Neubauer chamber. Haematocrit (Ht) and hemoglobin (Hb; SPINREACT kit, ref. 1001230, Spain) were also evaluated, and the mean corpuscular volume (MCV), mean corpuscular hemoglobin (MCH) and mean corpuscular hemoglobin concentration (MCHC) were calculated as previously described (31). Blood smears were done with 3 µL of lightly homogenized blood, air-dried, and fixed for 1 min in formol-ethanol (10% of 37% formaldehyde in absolute ethanol). The identification of neutrophils was performed using the peroxidase detection method outlined by Afonso et al. (32). Blood smears were then stained using Wright’s stain (Haemacolor; Merck). Slides were examined under oil immersion 100 × objective (final magnification of 1,000 ×), and 200 leucocytes were counted and categorized, based on their morphology and staining characteristics, as thrombocytes, lymphocytes, monocytes, and neutrophils. The total number of WBCs was multiplied by the percentage of each cell population to calculate the number of cells *per* µL of blood.

### 2.6 Collection of peritoneal exudates

The peritoneal cells were collected according to the procedure first described for mice by Silva et al. (33) and posteriorly adapted for fish by Afonso et al. (34). Briefly, after blood collection from the caudal vein, 2 mL of sterile HBSS supplemented with 30 units of heparin mL^−1^ was injected into the peritoneal cavity. Following that, the peritoneal region was gently massaged to spread the peritoneal cells in the injected HBSS, and the i.p. injected HBSS with the resuspended cells was then collected. A volume of 10 µL was plated in MA, followed by incubation for 72 h at 25°C to evaluate the presence of viable *T. maritimum*. Total peritoneal cell counts were performed with a hemocytometer. The Cytospin preparations were performed using a THARMAC Cellspin device and were stained, as mentioned before, for blood smears. The peritoneal exudates were differentially counted and identified as lymphocytes, macrophages, and neutrophils. The percentage of each cell type was determined after counting a minimum of 200 cells per slide. The obtained counting values were then used to calculate the number of each leucocyte type *per* peritoneal cavity.

### 2.7 Bacterial DNA Extraction and PCR analysis

Bacterial colonies grown on MA plates inoculated with blood and peritoneal exudates were re-plated in MB and grown at 25 °C for 48-72 h. DNA was extracted from the cultures using NZY Tissue gDNA Isolation kit (NZYTech, Lisbon, Portugal), following the manufacturer’s instructions, and maintained at –20°C until use. DNA extracted from a pure culture of the *T. maritimum* strain ACC13.1 and sterile distilled water were used as positive and negative controls, respectively. Then, a PCR was performed according to Avendaño-Herrera et al. (35) using the species-specific primer set MAR1 (5’-AATGGCATCGTTTTAAA-3’) and MAR2 (5’-CGCTCTCTGTTGCCAGA-3’) (36) designed against 16S ribosomal gene. The PCR amplification was performed with the NZYTaq II Green Master Mix (NZYTech, Lisbon, Portugal). The PCR reaction was done in a Veriti DX 96-well Thermal Cycler (Applied Biosystems, Foster City, CA, USA). The samples were denatured at 94°C for 2 min, followed by 30 cycles of 94°C for 2 min, 45°C for 90 s, and 72°C for 2 min. Afterwards, the samples were maintained at 4°C. The PCR products were analyzed by 2% agarose gel electrophoresis for 50 min at 100 V in TAE Buffer, pH 8 (NZYTech, Lisbon, Portugal) using NZYDNA Ladder I (NZYTech, Lisbon, Portugal) as a molecular size marker. DNA bands were visualized with GreenSafe Premium (0.03 µL mL^-1^) (NZYTech, Lisbon, Portugal) and images were obtained with Gel Doc XR+ Image Lab Software (BioRad).

### 2.8 Proteomic analysis

The ECPs sample used for the i.p. challenge was processed for proteomic analysis following the solid-phase-enhanced sample-preparation (SP3) protocol and enzymatically digested with Trypsin/LysC as previously described (37). Protein identification and quantitation were performed by nanoLC-MS/MS equipped with a Field Asymmetric Ion Mobility Spectrometry - FAIMS interface. This equipment is composed of a Vanquish Neo liquid chromatography system coupled to an Eclipse Tribrid Quadrupole, Orbitrap, Ion Trap mass spectrometer (Thermo Scientific, San Jose, CA). Briefly, 250 ng of peptides of each sample were loaded onto a trapping cartridge (PepMap Neo C18, 300 μm × 5 mm i.d., 174500, Thermo Scientific, Bremen, Germany). Next, the trap column was switched in-line to a μPAC Neo 50 cm column (COL-nano050NeoB) coupled to an EASY-Spray nano flow emitter with 10 μm i.d. (ES993, Thermo Scientific, Bremen, Germany). A 130 min separation was achieved by mixing A: 0.1% FA and B: 80% ACN, 0.1% FA with the following gradient at a flow of 750 µL min^-1^: 0.1 min (1% B to 4% B) and 1.9 min (4% B to 7% B). Next, the flow was reduced to 250 µ L min^-1^ with the following gradient: 0.1 min (7.0 to 7.1% B), 80 min (7.1% B to 22.5% B), 30 min (22.5% B to 40% B), 8 min (40% B to 99% B) and 9.9 min at 99% B. Subsequently, the column was equilibrated with 1% B. Data acquisition was controlled by Xcalibur 4.6 and Tune 4.0.4091 software (Thermo Scientific, Bremen, Germany).

MS results were obtained following a Data Dependent Acquisition - DDA procedure. MS acquisition was performed with the Orbitrap detector at 120 000 resolution in positive mode, quadrupole isolation, scan range (*m/z*) 375-1500, RF Lens 30%, standard AGC target, maximum injection time was set to auto, 1 microscan, data type profile and without source fragmentation. FAIMS mode: standard resolution, total carrier gas flow: static 4 L min^-1^, FAIMS CV: -45, -60 and -75 (cycle time, 1 s). Internal Mass calibration: Run-Start Easy-IC. Filters: MIPS, monoisotopic peak determination: peptide, charge state: 2-7, dynamic exclusion 30s, intensity threshold, 5.0e3. MS/MS data acquisition parameters: quadrupole isolation window 1.8 (m/z), activation type: HCD (30% CE), detector: ion trap, IT scan rate: rapid, mass range: normal, scan range mode: auto, normalized AGC target 100%, maximum injection time: 35 ms, data type centroid.

The raw data was processed using the Proteome Discoverer 3.0.1.27 software (Thermo Scientific) and searched against the UniProt database for the *T. maritimum* NCIMB2154 Proteome (2022_03 with 2,844 entries). A common protein contaminant list from MaxQuant was also included in the analysis. The Sequest HT search engine was used to identify tryptic peptides. The ion mass tolerance was 10 ppm for precursor ions and 0.5 Da for fragment ions. The maximum allowed missing cleavage sites was set to two. Cysteine carbamidomethylation was defined as constant modification. Methionine oxidation, deamidation of glutamine and asparagine, peptide terminus glutamine to pyroglutamate, and protein N-terminus acetylation, Met-loss, and Met-loss+acetyl were defined as variable modifications. Peptide confidence was set to high. The processing node Percolator was enabled with the following settings: maximum delta Cn 0.05; target FDR (strict) was set to 0.01, and target FDR (relaxed) was set to 0.05, validation based on *q*-value. Protein label-free quantitation was performed with the Minora feature detector node at the processing step. Precursor ions quantification was performed at the consensus step with the following parameters: unique plus razor peptides were considered, precursor abundance based on intensity, and normalization based on total peptide amount.

Raw data hits from the single ECPs sample were filtered using coverage above 30%, unique peptides above 3 and a SEQUEST HT score greater than 100; the obtained hits were automatically assigned the corresponding GO terms using the UniProt tool ID Mapping (https://www.uniprot.org/id-mapping, accessed 8^th^ Feb 2024).

### 2.9 Innate immune parameters

#### 2.9.1 Antiprotease and protease activities

The antiprotease activity was calculated using Ellis (38) methodology, modified for 96-well microplates. Briefly, 10 µL of plasma was incubated with 10 µL of trypsin solution (5 mg mL^-1^ in 0.5% NaHCO_3_, pH 8.3) (Sigma, USA) for 10 min at 22°C in microtubes. Following the initial incubation, 125 µL of azocasein (20 mg mL^-1^ in 0.5% NaHCO_3_, pH 8.3) and 100 µL of phosphate buffer (115 mM NaH_2_PO_4_, pH 7.0) were added. This step was followed by another one-hour incubation at 22°C in the dark with agitation. Next, 250 µL of 10% cold trichloroacetic acid (TCA) was added to the mixture, incubated for 30 min at 22°C, and centrifuged at 10,000 *× g* for 5 min at room temperature (RT). Lastly, 100 µL was transferred to a 96-well plate containing 100 µL of 1 N NaOH *per* well, in duplicate, and the absorbance read at 450 nm in a Synergy HT microplate reader. The absorbance obtained with phosphate buffer, instead of plasma, was used as a reference, and the percentage of trypsin activity was calculated as follows: 100 − ((sample absorbance/reference absorbance) × 100).

To determine protease activity, the same protocol was applied, without the initial incubation with trypsin and the incubation with azocasein and phosphate buffer was done for 24 h instead of 1 h, in constant agitation. Plasma was replaced by trypsin (5 mg mL^-1^) as a positive control or by phosphate buffer as a negative control. The percentage of trypsin activity compared to the positive control was determined according to (sample absorbance/positive control absorbance) × 100.

#### 2.9.2 Peroxidase

Plasma peroxidase activity was assessed using the technique described by Quade and Roth (39). In triplicates, 5 µL of plasma was diluted in 145 µL of HBSS without Ca^+2^ and Mg^+2^ (Cytiva, USA) in flat-bottom 96-well plates. Next, 50 µL of 20 mM 3,3’,5,5’- tetramethylbenzidine hydrochloride (TMB; Sigma, USA) was added to each well. The reaction was stopped after 2 min by adding 50 µL of 2 M sulphuric acid, and the absorbance was measured at 450 nm (Synergy HT microplate reader). Peroxidase activity (units mL^-1^ plasma) was calculated by defining one unit of peroxidase as the amount needed to produce an absorbance change of 1 at 450 nm.

#### 2.9.3 Lysozyme activity

Lysozyme activity was assessed using a turbidimetric assay mentioned by Costas et al. (40). Initially, a suspension of *Micrococcus lysodeikticus* (0.5 mg mL^-1^ in 0.05 M sodium phosphate buffer, pH 6.2) was prepared. In triplicates, 15 µL of plasma was added to a microplate and 250 µL of the previous suspension was pipetted to give a final volume of 265 µL. The reaction was carried out at 25°C, and the absorbance (450 nm) was measured after 0.5 and 5 min in a Synergy HT microplate reader. A standard curve was created using lyophilised hen egg white lysozyme (Sigma, USA) serially diluted in sodium phosphate buffer (0.05 M, pH 6.2). This standard curve was then used to calculate the amount of lysozyme in each sample.

#### 2.9.4 Bactericidal activity

Bacteria (*T. maritimum* ACC13.1) were grown on MA at 25°C for 48 h and resuspended in MB at a concentration of 1.6 × 10^8^ CFU mL^-1^ by determining the turbidity at 600 nm (Synergy HT microplate reader) and using the previously mentioned growth curve: y = 2 × 10^8^x + 4 × 10^7^. The bactericidal activity of plasma was subsequently assessed using a method similar to the one outlined by Graham et al. (41) but with certain adjustments, as described by MaChado et al. (31). In a U-shaped 96-well plate, 20 µL of plasma was added in duplicates, and as a negative control, MB was added to the wells instead of plasma. To each well, 20 µL of bacteria was added to the plate and incubated for 2.5 h at 25°C. Then, 25 µL of 3-(4, 5 dimethyl-2-yl)-2,5-diphenyltetrazolium bromide (MTT, 1 mg mL^-1^; Sigma) was added to the wells, and the plate was incubated again for 10 min at 25°C. Plates were centrifuged at 2,000 *× g* for 10 min, and formazan precipitate was dissolved with 200 µL of dimethyl sulfoxide (Sigma, USA) and quantified by measuring the absorbance at 560 nm (Synergy HT microplate reader). In this method, the difference between the formazan formed in the samples and the negative control (100% viability) enables calculating both viable bacteria and the percentage of non-viable bacteria in each sample.

#### 2.9.5 Nitrite concentration

Compounds such as nitrite and nitrate, which are endogenously produced as oxidative metabolites of the messenger molecule NO, are considered indicative of NO production (42). Thus, to indirectly access the nitric oxide (NO) concentration in plasma, a Nitrite/Nitrate colorimetric kit (Roche, 11746081001, Germany) was utilised, according to the manufacturer’s instructions. The samples were diluted 1:10 in distilled H_2_O, and the concentrations were expressed as µM.

### 2.10 Oxidative stress biomarkers

Liver tissue was homogenized 1/10 (w/v) in potassium phosphate buffer (0.2 M, pH 7.4). For lipid peroxidation (LPO) assessment, 200 µL of the homogenised mixture was transferred to a microtube containing 4 µL of 4% BHT (2,6-Di-tert-butyl-4-methylphenol) in methanol. For the assessment of superoxide dismutase and catalase activities, each volume of tissue homogenate was added to a volume of potassium phosphate buffer (0.2 M, pH 7.4), and centrifuged at 10,000 *× g* for 20 min at 4°C. The supernatants were collected and maintained at −80°C. Protein concentration was determined using Pierce™ BCA Protein Assay kit (Thermo Fischer Scientific USA), with bovine serum albumin as standard, according to the manufacturer’s guidelines. For superoxide dismutase and catalase activities, the homogenized liver was diluted to reach a final protein concentration of 0.3 mg mL^-1^.

LPO was calculated using the procedure outlined by Bird and Draper (43) with some modifications (44). Therefore, 100 µL of 100% TCA was added to 204 µL of liver homogenate with 4% BHT together with 1 mL of 0.73% thiobarbituric acid solution [in 60 mM Tris–HCl, pH 7.4, 0.1 mM diethylenetriaminepentaacetic acid (DTPA)]. Samples were centrifuged at 15,000 × *g* for 5 minutes after being incubated for 1 hour at 100°C in a kiln. Afterward, 200 µL of supernatant was transferred to a 96-well plate in triplicates, and the absorbance was measured at 535 nm. The LPO was expressed as nmol of thiobarbituric acid reactive substances (TBARS) generated *per* g of wet tissue.

Catalase activity was assessed by measuring the decrease in absorbance through the consumption of H_2_O_2_, as defined by Claiborne (45), but by adapting the techique to microplates, as mentioned by Rodrigues et al. (46). A sample of 10 µL was put in triplicates onto a UV light microplate along with 150 µL of 30% H_2_O_2_ and 140 µL of 50 mM potassium phosphate buffer (pH 7.0). The absorbance was measured at 240 nm for 2 min. The catalase activity was quantified using the H_2_O_2_ molar extinction coefficient at 240 nm of 40 M cm^-1^, expressed in U *per* mg of protein.

Using cytochrome C method with xanthine/xanthine oxidase, superoxide dismutase (SOD) activity was quantified in accordance with the methodology described by Almeida et al. (47). In triplicates, a volume of 50 µL of each sample was transferred to a microplate. Then, 200 µL of a reaction solution, which contained 50 mM potassium phosphate buffer (pH 7.8) containing 1 mM Na-EDTA, 0.7 mM xanthine, and 0.03 mM cytochrome C, was added. Immediately after, 50 µL of 0.03 U mL^-1^ xanthine oxidase with 0.1 mM Na-EDTA was also put onto the microplate. The absorbance was measured at 550 nm (Synergy HT microplate reader) at 20 s intervals for 3 min. Activity is described as units of SOD *per* mg of protein. One unit of activity was defined as the quantity of enzyme necessary to produce a 50% inhibition of the cytochrome C reduction rate.

The reduced (GSH): oxidized (GSSG) glutathione ratio was quantified using the microplate assay for the GSH/GSSG commercial kit (Oxford Biomedical Research, UK), as previously outlined by Hamre et al. (48). This method depends on the quantitative determination at 412 nm of the total amount of glutathione (GSH + GSSG) and GSSG (49). In short, the determination of GSSG is achieved by adding a thiol scavenger (N-ethylmaleimide pyridine derivative solution, Oxford Biomedical Research, UK), that reacts with GSH to form a stable complex, removing the GSH before the quantification of GSSG, without inhibiting glutathione reductase (GR) activity. Adding glutathione reductase, the available GSSG is reduced to GSH, reacting with 5,5’-dithiobis-2-nitrobenzoic acid (DTNB), which allows the quantification of pre-existent GSSG. The reaction rate is proportional to the GSH and GSSG concentration. The GSH/GSSG Ratio is calculated as follows: (GSH_t_ – 2GSSG)/GSSG.

### 2.11 Gene expression analysis

Head-kidney tissue (n = 9 *per* treatment) was weighted (up to 20 mg of tissue), placed in 200 µL of chilled homogenization buffer and homogenized in Precellys Evolution homogenizer at 6,000 *× g* (2 × 20 s, 4°C) using the reagents provided by the Maxwell^®^ RSC simplyRNA Tissue Kit (Promega, USA). After adding 200 µL of lysis buffer to the samples, all total RNA isolations were performed by Maxwell^®^ RSC (Cat. # AS4500).

RNA samples were quantified, and purity was evaluated by spectrophotometry using DeNovix DS-11 FX (Wilmington, DE, USA) with absorbance ratios at 260 nm/280 nm of 2.1–2.2. First-strand cDNA was synthesized, and samples were standardized with the NZY First-Strand cDNA Synthesis Kit (NZYTech, Lisbon, Portugal), which was stored at -80°C. The Veriti DX 96-well Thermal Cycler (Applied Biosystems, Foster City, CA, USA) was utilized for reverse transcription. Real-time Quantitative PCR was performed with CFX384 Touch Real-Time PCR Detection System (Biorad, Hercules, CA, USA) using 4.4 µL of diluted cDNA mixed with 5 µL of iTaq Universal SYBR Green Supermix^®^ (Biorad, Hercules, CA, USA) and 0.3 µL (10 µM) of each primer, resulting in a final volume of 10 µL. Primers were designed with NCBI Primer Blast Tool and IDT OligoAnalyzer ToolTM to amplify European sea bass genes of interest. The known qPCR requirements were taken into account. The template sequences used for the primer’s design were obtained from both NCBI and the databases dicLab v1.0c sea bass genome (50). Using serial 2-fold dilutions of cDNA, the efficiency of each primer pair was assessed by calculating the slope of the regression line of the cycle thresholds (Ct) vs. the relative concentration of cDNA. The respective melting curves were analyzed to ensure no amplification of primer dimers. The standard cycling conditions were initial denaturation at 95 °C for 10 min, followed by 40 cycles of two steps (denaturation at 95 °C for 15 s followed by primer annealing temperature for 1 min), 95 °C for 1 min followed by 35 s at the annealing temperature, and 95 °C for 15 s. The reactions were run in duplicates, and target gene expression was normalized using the geometric mean of elongation factor 1β (*ef1β*) and ribosome 40s subunit (*40s*), calculated according to the Pfaffl method (51).

The accession numbers, primer efficiencies, annealing temperatures, amplicon length, and primer sequences are detailed in **Table 1**.

**Table 1:** Immune-related genes analyzed by Real-time PCR.

Gene	Acronym	Accession number	Efficiency ^a^	Annealing (°C)	Amplicon (bp)	Primer sequence (5’-3’)
Elongation factor 1-beta	*ef1b*	AJ866727.1	107.6	60	144	F: AACTTCAACGCCCAGGTCAT R: CTTCTTGCCAGAACGACGGT
40s Ribosomal protein	*40s*	HE978789.1	109.7	60	79	F: TGATTGTGACAGACCCTCGTG R: CACAGAGCAATGGTGGGGAT
Interleukin 1-beta	*il1β*	AJ269472.1	111.7	60	105	F: AGCGACATGGTGCGATTTCT R: CTCCTCTGCTGTGCTGATGT
Interleukin 6	*il6*	AM490062.1	102.8	60	81	F: AGGCACAGAGAACACGTCAAA R: AAAAGGGTCAGGGCTGTCG
Interleukin 8	*il8*	AM490063.1	106.3	60	140	F: CGCTGCATCCAAACAGAGAGCAAAC R: TCGGGGTCCAGGCAAACCTCTT
Interleukin 10	*il10*	AM268529.1	100.9	55	164	F: ACCCCGTTCGCTTGCCA R: CATCTGGTGACATCACTC
Interleukin 34	*il34*	DLAgn_00164750	99.8	60	129	F: GGAAATACGCTTCAGGGATG R: GGCACTCTGTCGGGTTCTT
Caspase 1	*casp1*	DQ198377.1	105.8	62	190	F: GTGTTTCAGATGCGGGAGGA R: ATTTAAGTTAACTCACCGGGGG
Tumor necrosis factor-alpha	*tnfα*	DQ070246.1	101.6	60	112	F: AGCCACAGGATCTGGAGCTA R: GTCCGCTTCTGTAGCTGTCC
Matrix metallopeptidase 9	*mmp9*	FN908863.1	105.8	57	166	F: TGTGCCACCACAGACAACTT R: TTCCATCTCCACGTCCCTCA
Chemokine CXC receptor 4	*cxcr4*	FN687464.1	90.9	57	171	F: ACCAGACCTTGTGTTTGCCA R: ATGAAGCCCACCAGGATGTG
Macrophage migration inhibitory factor	*mif*	AY423555.2	97.6	62	88	F: GCTCCCTCCACAGTATTGGCAAGAT R: TTGAGCAGTCCACACAGGAGTTTAGAGT
Macrophage colony-stimulating factor 1 receptor	*mcsfr*	FN582353	104.4	55	76	F: ATGTCCCAACCAGACTTTGC R: GGCTCATCACACACTTCACC
Major histocompatibility complex II antigen beta chain	*mhcII*	AM113468.1	108.9	55	81	F: ATCCCTCCATGTTGGTCTGC R: CTTCCTGTCCGTCTCTGAGC
Heat shock protein 70	*hsp70*	AY423555.2	104.9	55	88	F: ACAAAGCAGACCCAGACCTTCACCA R: TGGTCATAGCACGTTCGCCCTCA
Hepcidin	*hamp1*	KJ890396.1	103.1	60	148	F: ACACTCGTGCTCGCCTTTAT R: TGTGATTTGGCATCATCCACG
Ferroportin	*fpn*	KU599935.1	109.7	60	161	F: GCTAGAGTTGGCCTGTGGTC R: GGGTTCGGAGCCAGTATCAC

a Efficiency of PCR reactions was calculated from serial dilutions of tissue RT reactions in the validation procedure.

### 2.12 Statistical analysis

Data were analyzed for normality and homogeneity of variance, and when necessary, outliers were removed. Gene expression data was Log-transformed before being statistically analyzed, and peritoneal cells *per* cavity, differential cell counts, and the GSH/GSSG ratio were Box-Cox transformed. The Student’s *t*-test was used to evaluate differences between the control (undisturbed) and each treatment (mock or ECPs) group for each time point. An analysis of variance (Two-way ANOVA) was applied, followed by an LSD test to evaluate statistically significant differences between time points and treatment (mock, BWO and BECPs groups) (interaction between factor time and treatment); to determine differences in time points or treatments (mock, BWO and BECPs groups), an analysis of variance (One-way ANOVA) was applied (no interaction between factor time and treatment), followed by Tukey’s *post hoc* test. The Student’s *t*-test was also used to evaluate differences between control (undisturbed) and treatment (mock, BWO, and BECPs) groups for each time point.

The significance level was set at 0.05 for all statistical tests. All calculations and statistical analyses were performed under the SPSS 29 program for Windows. Results were presented as the mean ± standard error of the mean (SEM). All graphs were designed with the Graph Pad Prism 8.01 Software.

## 3 Results

### 3.1 Protein composition of ECPs

Analysis of the *T. maritimum*’s ECPs revealed a complex protein profile, with band sizes ranging from 20 to over 250 kDa (**Supplementary Figure S1**).

To identify the proteins present in the *T. maritimum*’s ECPs, these were analysed by NanoLC-MS/MS. A total of 744 non-redundant proteins were identified in the concentrated ECPs, which would represent approximately 11.62% of the theoretical proteome of *T. maritimum* NCIMB 2154 (DOI:10.6084/m9.figshare.26014573). A list of the filtered hits (coverage above 30%, unique peptides superior to 3 and SEQUEST HT score greater than 100) is presented in **Supplementary Table S1**. For a better interpretation of the results, the hits were classified according to their associated GoTerm using the UniProt ID mapping platform (https://www.uniprot.org/id-mapping, accessed on 8^th^ Feb 2024). The obtained protein hits were related to important biological processes, such as proteolysis, cell adhesion and carbohydrate metabolic processes (**Supplementary Table S1**). Some of the most abundant proteins were lipoproteins, with several others being predicted proteins secreted by the T9SS, as described by (21), such as multimodular sialidase/sialate O-acetylesterase/sialidase (MARIT_2686) and probable M14 family carboxypeptidase (MARIT_2507), containing a C-terminal secretion signal (**Supplementary Table S1**). Other proteins were related to iron acquisition strategies, like iron-regulated protein imelysin family lipoprotein (MARIT_1664) and heme binding lipoprotein HmuY-family (MARIT_2477), or related to mechanisms to face up oxidative stress scenarios, like superoxide dismutase (MARIT_3105), thioredoxin (MARIT_2619) and alkyl hydroperoxide reductase (MARIT_0947) (**Supplementary Table S1**). Outer membrane and TonB-related proteins like OmpA family protein (MARIT_2995), TonB-dependent outer membrane receptor SusC/RagA family (MARIT_2376) and TonB-dependent receptor (MARIT_0214) were also identified (**Supplementary Table S1**). Components of the gliding motility machinery described for *Flavobacterium*, by Gorasia et al. (52), were also identified in *T. maritimum*’s ECPs, such as PorU (MARIT_0895), PorV (MARIT_0894), GldM (MARIT_0756), GldN (MARIT_0757) and SprD (MARIT_1320) (DOI:10.6084/m9.figshare.26014573). Additionally, other proteins related to T9SS were identified, including adhesin SprC (MARIT_1318), SprA (MARIT_2960), SprT (MARIT_0579) and SprF (MARIT_1793), (DOI:10.6084/m9.figshare.26014573).

### 3.2 Percentage survival

**Figure 1:**
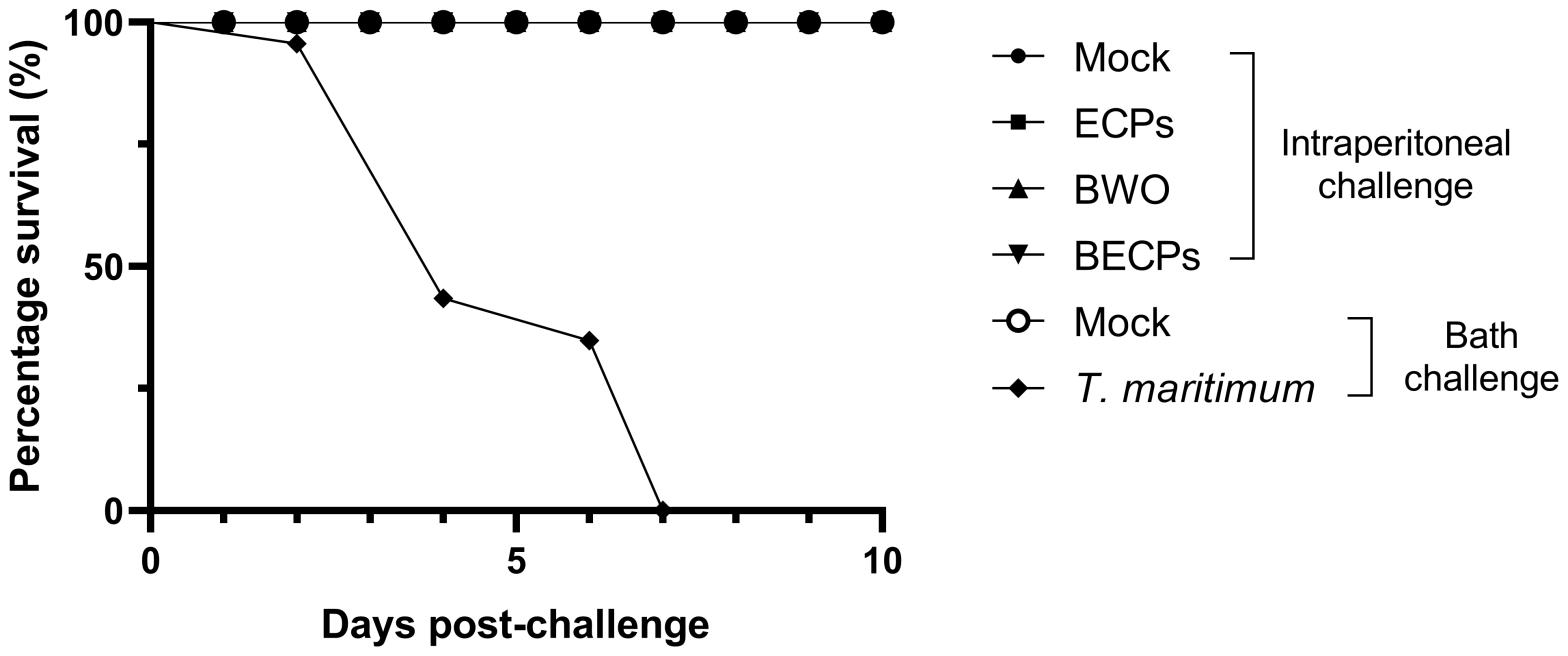
Percentage survival (%) after intraperitoneal injection of 100 µL MB (Mock), 100 µL *T. maritimum*’s ECPs (ECPs), 100 µL MB containing 5.5 x 10^5^ CFU *T. maritimum* without ECPs (BWO) or 100 µL MB containing 5.5 × 10^5^ CFU *T. maritimum* with ECPs (BECPs) (n = 21 *per* group) or after bath challenge with MB (Mock) or 5.5 × 10^5^ CFU mL^-1^ *T. maritimum* without ECPs (n = 12 *per* group).

A 100% survival was observed in fish challenged i.p. with ECPs or with bacteria with or without ECPs (**Figure 1**). However, a percentage survival of 0% was obtained at day 7 for the fish challenged by bath (n = 12 *per* treatment, X^2^ < 0.0001) (**Figure 1**).

### 3.3 Re-isolation of T. maritimum from blood and peritoneal exudates

All peritoneal exudate samples collected from fish i.p. injected with BWO and BECPs at 3 and 6 h post-challenge presented bacterial growth in MA plates (**Table 2**). However, only 2/12 and 3/12 blood samples collected at 3 h post-challenge from fish injected with BWO and BECPs, respectively, were positive for bacterial growth (**Table 2**). At 6 h post-challenge, no bacterial growth was recorded for the blood from fish injected with BWO, while 11/12 samples from the BECPs group had bacterial growth (**Table 2**). After this sampling time point, no bacterial growth was seen in the peritoneal exudates or blood (**Table 2**). As expected, the blood and peritoneal exudates from undisturbed controls and mock-treated fish did not show bacterial growth (**Table 2**). Bacterial cultures recovered from inoculated fish showed *Tenacibaculum-*like characteristics, with pale/translucent colonies with uneven edges, flat and adherent between them and PCR analysis confirmed that they corresponded to *T. maritimum* (amplification of a single product with the expected size) (**Supplementary Figure S2**).

**Table 2:** Bacterial growth in aseptically collected peritoneal exudates (PE) and blood from undisturbed fish (Control) or from fish i.p. challenged with 100 µL MB (Mock), 100 µL *T. maritimum*’s ECPs (ECPs), 100 µL MB containing 5.5 × 10^5^ CFU *T. maritimum* without ECPs (BWO) or 100 µL MB containing 5.5 × 10^5^ CFU *T. maritimum* with ECPs (BECPs) (n = 12 *per* treatment).

	0 h		3 h		6 h		24 h		48 h	
	PE	Blood	PE	Blood	PE	Blood	PE	Blood	PE	Blood
**Control**	0/12	0/12	–	–	–	–	–	–	–	–
**Mock**	**-**	–	0/12	0/12	0/12	0/12	0/12	0/12	0/12	0/12
**ECPs**	**-**	–	0/12	0/12	0/12	0/12	0/12	0/12	0/12	0/12
**BWO**	**-**	–	12/12	2/12	12/12	0/12	0/12	0/12	0/12	0/12
**BECPs**	**-**	–	12/12	3/12	12/12	11/12	1/12	0/12	0/12	0/12

### 3.4 Peritoneal cell numbers and haematological parameters

**Figure 2:**
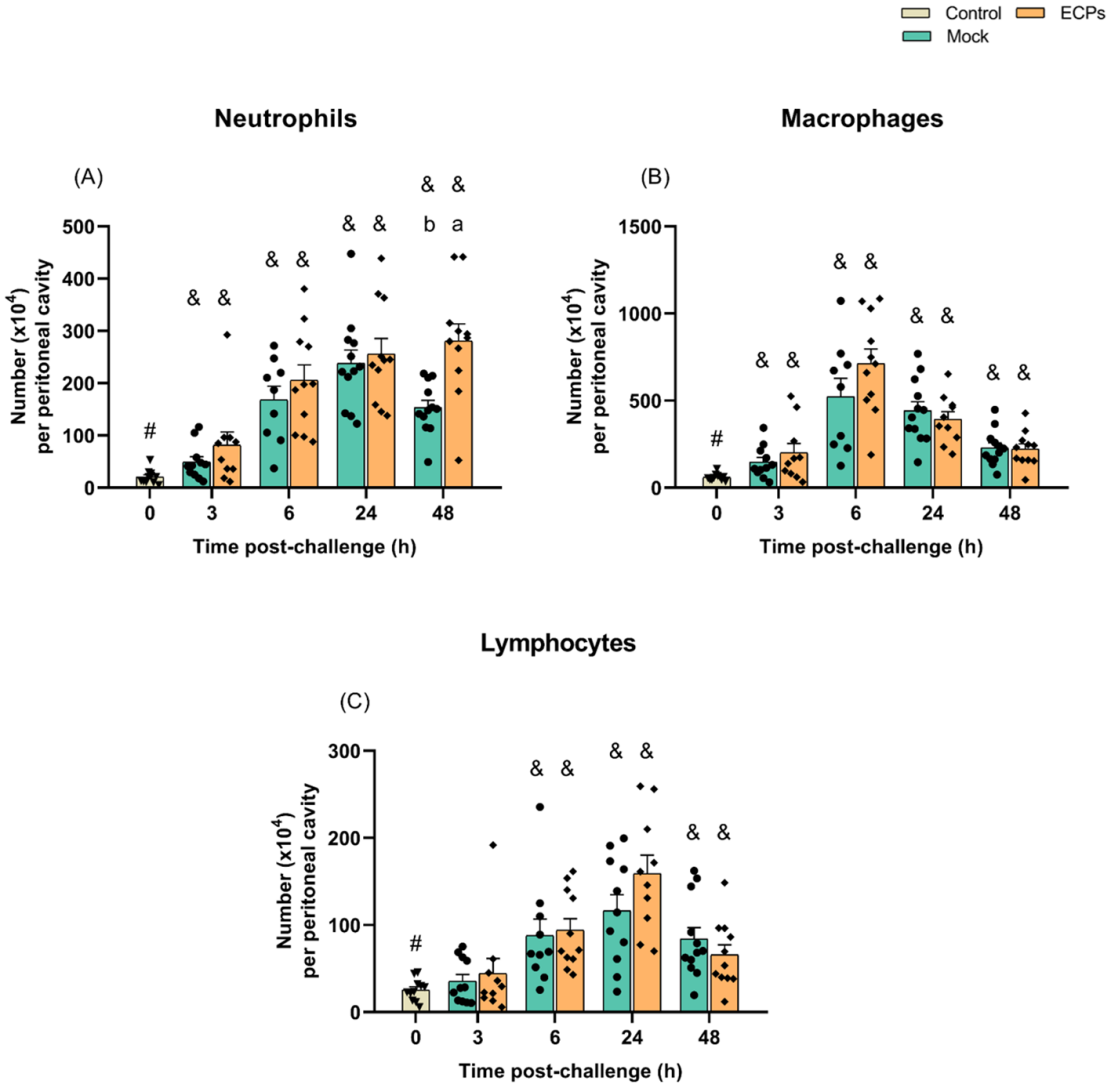
Numbers of neutrophils **(A)**, macrophages **(B)** and lymphocytes **(C)** in the resting peritoneal cavity (Control – grey column) or in the peritoneal cavity of European sea bass i.p. challenged with 100 µL MB (Mock – blue columns) or 100 µL *T. maritimum*’s ECPs (ECPs – orange columns). Data are expressed as mean ± SEM (n = 12 *per* treatment). Different lowercase letters stand for significant differences between treatments among time points and different symbols (&) represent significant differences between the control group (undisturbed - #) and the remaining groups (*Student’s* t-test; *p* ≤ 0.05).

No significant differences in the numbers of peritoneal cells (neutrophils, macrophages and lymphocytes) were observed after i.p. injection of ECPs when compared with the mock (**Figure 2**). In what concerns the systemic response, a decrease in the total number of WBC was observed both in fish challenged with ECPs or MB (mock) when compared to the undisturbed control group, but no significant differences between ECPs or mock groups were recorded (**Supplementary Table S2**). Despite the lack of differences in total WBC, the number of circulating neutrophils was significantly higher at 3 and 6 h post-challenge in the ECPs’ group, when compared to the mock, decreasing afterwards in the 48 h sampling point (**Supplementary Table S2**). In contrast, no differences were recorded for monocytes, lymphocytes and thrombocytes (**Supplementary Table S3**). Also, injection of ECPs did not affect the total number of RBC (**Supplementary Table S2**) and failed to induce significant differences in haemoglobin concentration, haematocrit and the haematological ratios of mean corpuscular volume, mean corpuscular haemoglobin and mean corpuscular haemoglobin concentration (**Supplementary Table S2**).

**Figure 3:**
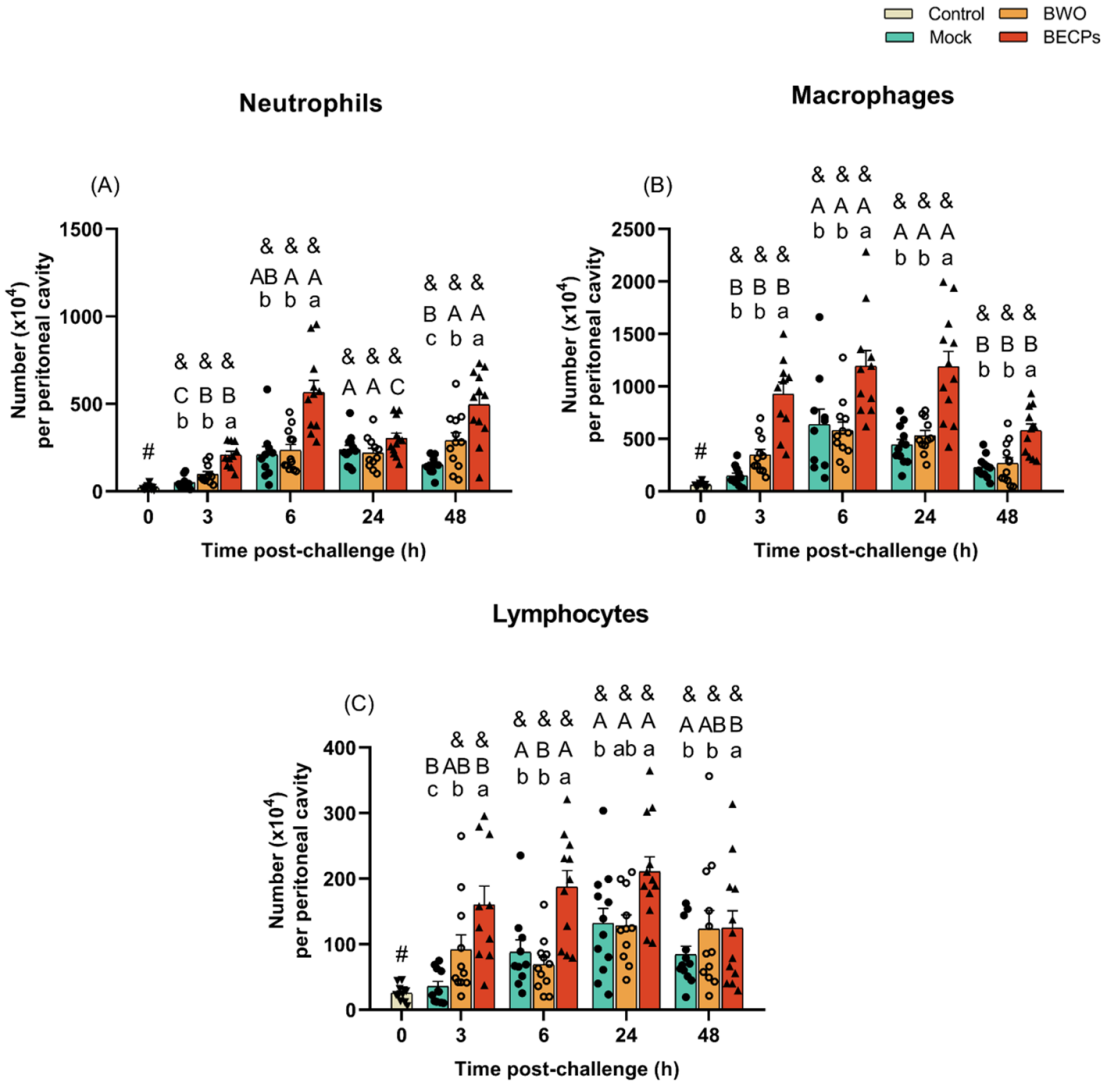
Numbers of neutrophils **(A)**, macrophages **(B)** and lymphocytes **(C)** in the resting peritoneal cavity (Control – grey column) or in the peritoneal cavity of European sea bass i.p. challenged with 100 µL MB (Mock – blue columns), 100 µL MB containing 5.5 × 10^5^ CFU *T. maritimum* without ECPs (BWO – orange columns) or 100 µL MB containing 5.5 × 10^5^ CFU *T. maritimum* with ECPs (BECPs – red columns). Data are expressed as mean ± SEM (n = 12 *per* treatment). Different lowercase letters stand for significant differences in treatments among each time point, while different capital letters indicate differences in time among the same treatment (Two-Way ANOVA for interaction between factors, followed by Tukey’s HSD or LSD for multiple comparisons, *p* ≤ 0.05). Different symbols (&) represent significant differences between the control group (undisturbed - #) and the different treatment groups (*Student’s* t-test; p ≤ 0.05).

In the experiments involving i.p. inoculation of bacteria, an increase in the numbers of peritoneal neutrophils, macrophages and lymphocytes was observed in the BECPs group, when compared to the mock. In contrast, no major changes in peritoneal cell numbers were recorded after injection of BWO, relative to the mock-challenged group (**Figure 3**).

At the systemic level, an abrupt decrease in total WBC was observed in the BWO and BECPs treatments at 3 h post-challenge when compared with the mock-challenged and control groups (**Supplementary Table S4**). Afterwards, the numbers of WBC in the BWO and BECPs groups remained low but similar to the numbers recorded in the mock-challenged fish (**Supplementary Table S4**).

The results of the differential cell counts revealed no significant differences between the mock and bacterial inoculated groups, for neutrophil and monocyte counts (**Supplementary Table S5**). Changes in the numbers of circulating lymphocytes and thrombocytes showed an emphasised decrease at 3 h in the BECPs and BWO groups, relative to mock-treated and control animals (**Supplementary Table S5**).

Afterwards, the levels of lymphocytes and thrombocytes remained low in BECPs and BWO groups but were not significantly different to the ones in mock-treated fish (**Supplementary Table S5**). A slight decrease in the RBCs counts was observed in the BECPs-treated group, when compared to the mock, with differences reaching significance at 3 and 6 h post-challenge, whereas no decrease was observed in the BWO group (**Supplementary Table S4**). Injection of BECPs also led to a decrease in the haematocrit, when compared to the BWO or mock treatments at 48 h (**Supplementary Table S4**). The remaining haematological ratios presented no major differences (**Supplementary Table S4**).

### 3.5 Innate humoral parameters

Regarding the innate humoral parameters, no major differences were observed between the mock and ECPs treatments (**Supplementary Table S6**). In what concerns the response to the injection of BWO or BECPs, an increase in the peroxidase activity was recorded at 48 h and a higher bactericidal activity was detected at 24 and 48 h, relative to the levels in mock-treated animals (**Supplementary Table S7**). The other parameters analysed did not show major differences (**Supplementary Table S7**).

### 3.6 Oxidative stress biomarkers

No changes in hepatic catalase activity were recorded in response to i.p. injection of ECPs when compared with mock treatment (**Supplementary Table S8**). The same was observed for the BWO and BECPs treatments (**Supplementary Table S9**). Superoxide dismutase activity in the liver significantly increased at 3 h post-challenge for the ECPs treatment, when compared to the mock and control groups, followed by a decrease at 6 h (**Supplementary Table S8**). The same response pattern was also obtained for the BWO and BECPs treatments, which showed increased superoxide dismutase activity at 3 h post-challenge when compared to the mock challenge group (**Supplementary Table S9**). No significant changes in lipid peroxidation were observed in fish i.p. injected with ECPs, when compared to mock-treated (**Supplementary Table S8**). The BECPs treatment led to an increase of lipid peroxidation as quickly as 3 h post-challenge, with a prolonged effect, since at 24 and 48 h post-challenge the values continued significantly high when compared to the mock group (**Supplementary Table S9**). Apart from an initial increase at 3 h post-challenge in the mock group in reduced:oxidized glutathione ratio when compared with the remaining inoculated groups (ECPs and bacterial ones), no major differences were seen among the remaining analysed oxidative stress parameters.

### 3.7 Gene expression analysis

Although a peritoneal response was not seen for the fish i.p. injected with *T. maritimum*’s ECPs, the gene expression profile of this group pointed to a systemic inflammatory response, quite similar to the one observed for the fish i.p. injected with bacteria plus ECPs.

**Figure 4:**
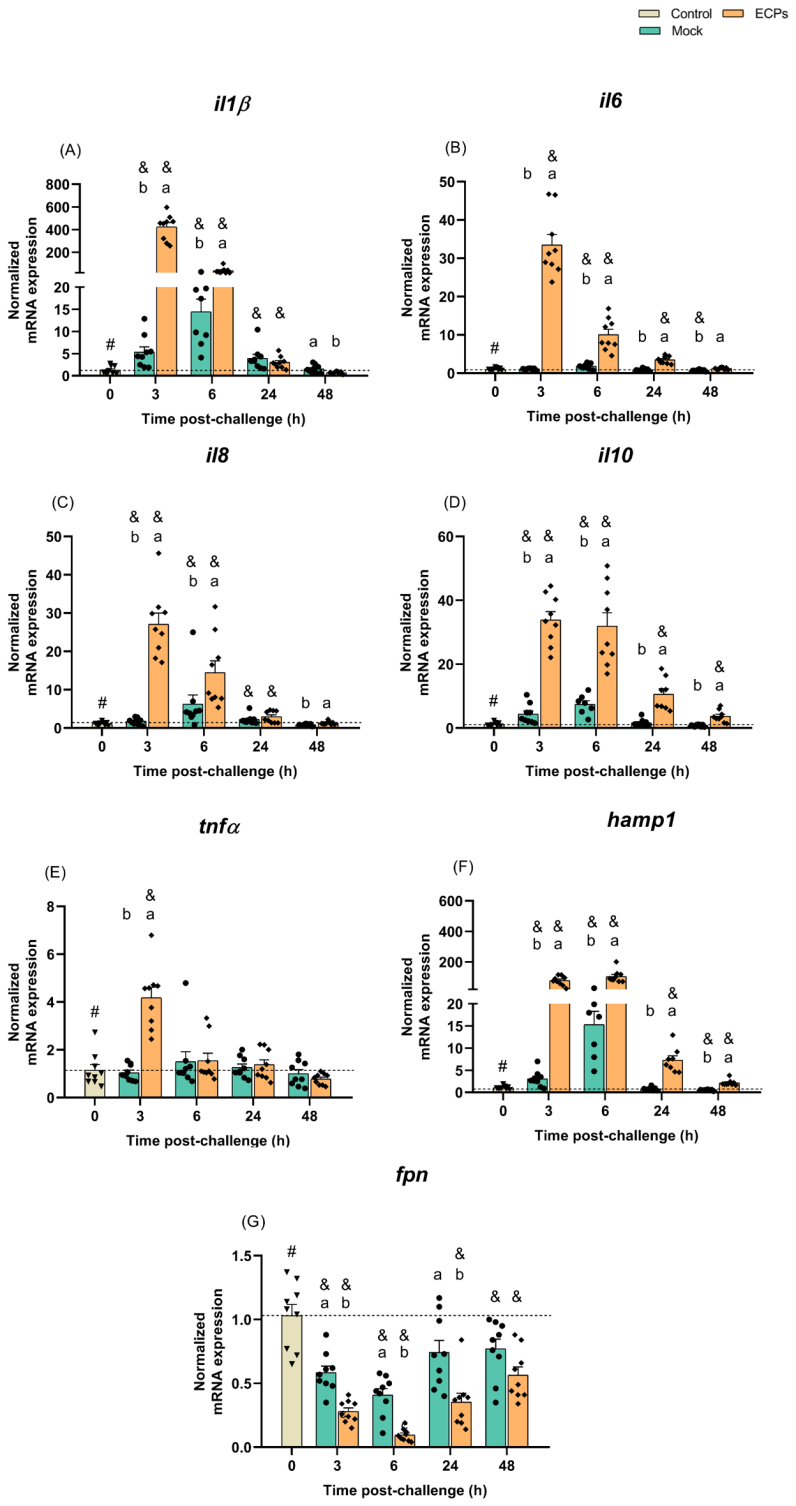
Quantitative expression of **(A)** *il1β*, **(B)** *il6*, **(C)** *il8*, **(D)** *il10*, **(E)** *tnfα*, **(F)** *hamp1* and **(G)** *fpn* (Control – grey column) in head-kidney of European sea bass i.p. challenged with MB (Mock – blue columns) or *T. maritimum*’s ECPs (ECPs – orange columns). Data are expressed as mean ± SEM (n=9 *per* treatment). Different lowercase letters stand for significant differences between treatments among time points and different symbols (&) represent significant differences between the control group (undisturbed - #) and the remaining groups (*Student’s* t-test; *p* ≤ 0.05).

**Figure 5:**
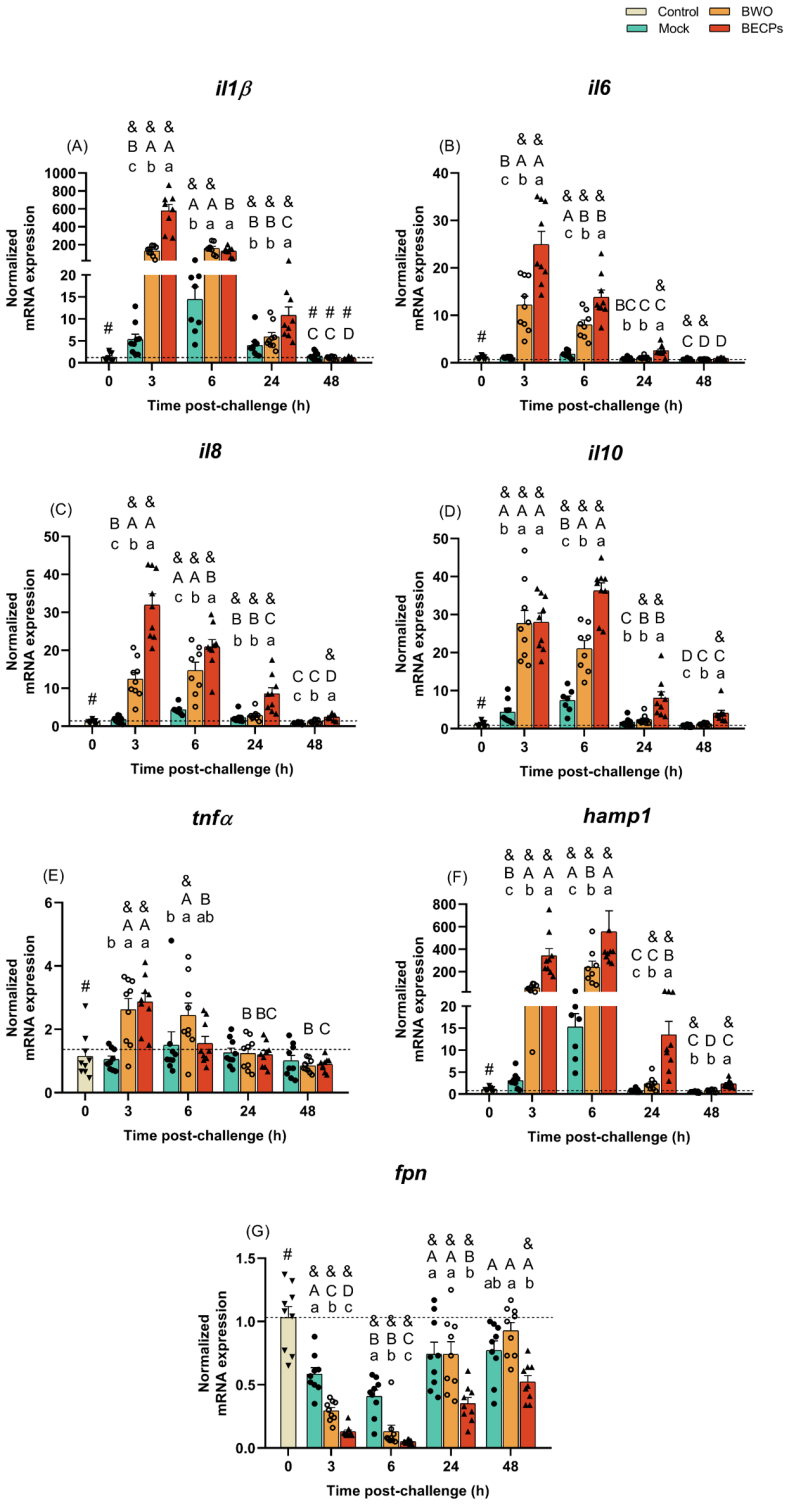
Quantitative expression of **(A)** *il1β*, **(B)** *il6*, **(C)** *il8*, **(D)** *il10*, **(E)** *tnfα*, **(F)** *hamp1* and **(G)** *fpn* (Control – grey column) for head-kidney of European sea bass i.p. challenged with MB (Mock – blue columns) or 5.5 × 10^5^ CFU *T. maritimum* without ECPs (BWO – orange columns) or 5.5 × 10^5^ CFU *T. maritimum* with ECPs (BECPs – red columns). Data are expressed as mean ± SEM (n=9 *per* treatment). Different lowercase letters stand for significant differences in treatments among each time point, while different capital letters indicate differences in time among the same treatment (Two-Way ANOVA for interaction between factors, followed by Tukey’s HSD or LSD for multiple comparisons, *p* ≤ 0.05). Different symbols (&) represent significant differences between the control group (undisturbed - #) and the different treatment groups (*Student’s* t-test; *p* ≤ 0.05).

A significant increase in interleukin 1 beta (*il1β*) expression was seen at 3 and 6 h post-challenge with ECPs compared to the control or mock groups (**Figure 4A**). At 3 h post-challenge, *il1β* expression in the ECPs-treated group was 80-fold higher than in mock challenge fish. After these sampling time points, the expression values of this inflammatory cytokine started to decrease, reaching values similar to the control group (**Figure 4A**). An identical response was observed for the fish i.p. injected with BECPs, recording an increase of *il1β* at 3 and 6 h, with a 305-fold increase at 3 h compared to the mock challenge group (**Figure 5A**). In this case, at 24 h post-challenge the immunogenic effect of BECPs treatment can still be seen, compared to the expression of BWO and mock treatments (**Figure 5A**). The interleukin 6 (*il6*), interleukin 8 (*il8*) and interleukin 10 (*il10*) responses in the course of this challenge were quite similar to the ones seen for *il1β*, for both ECPs and BECPs. For *il6* an exacerbated expression (an almost 38-fold increase regarding the mock group) was seen at 3 h post-challenge, followed by a decrease in the following time point (**Figure 4B**). The BWO and BECPs treatments also presented an increased expression at 3 and 6 h post-challenge for this cytokine when compared with the control and mock groups (**Figure 5B**). As previously mentioned, *il8* showed an identical response to *il1β*, for the ECPs group, as well as for the BECPs (**Figures 4**, **5C**). The anti-inflammatory cytokine *il10* presented an increased expression at 3 and 6 h post-challenge, reaching identical expression values for both sampling points in the ECPs treatment, when compared to control and mock fish (**Figure 4D**). The ECPs group remained different from the mock group until 48 h (**Figure 4D**), indicating a slightly sustained immune response. A similar type of kinetics was seen for *il10* for the bacteria injected groups, since at 3 h post-challenge no differences were recorded between BWO and BECPs treatments (**Figure 5D**), however, at 6 h post-challenge the BECPs group reached its maximum expression value and BWO group started to decrease (**Figure 5D**). Again until 48 h differences were recorded between the mock and BECPs groups (**Figure 5D**). Injection of ECPs induced the expression of tumor necrosis factor-alpha (*tnfα*) at 3 h post-challenge, with a 4-fold increase when compared to control and mock groups, followed by a decrease near basal levels afterwards (**Figure 4E**). The BECPs and BWO treatments also induced upregulation of *tnfα* expression at this time point, with an almost 3- and 2.4-fold increase, respectively, regarding the mock and control groups (**Figure 5E**). Afterwards, the expression decreased in both groups, although slower in BWO, reaching basal levels at 24 and 48 h post-challenge (**Figures 4**, **5E**).

Usually, the expression of IL-34 correlates with the expression of pro-inflammatory cytokines (e.g. IL1β and TNFα), but in this case, the values of expression of this cytokine in all i.p. injected groups remained low when compared to the control group. Initially, at 3 and 6 h post-challenge, mock and ECPs groups presented similar values. However, at 24 and 48 h, the expression of *il34* in the ECPs group was lower, when compared to the mock (**Supplementary Table S10**). A similar response was observed after injection of BECPs, with decreased *il34* expression at 24 h and 48 h, when compared to the mock group (**Supplementary Table S11**).

The expression of the chemokine receptor *cxcr4* was also downregulated in the ECPs group at 3 and 6 h post-challenge when compared to control and mock (**Supplementary Table S10**); the same kind of pattern was observed for BWO and BECPs groups at 3 and 6 h. However, *cxcr4* expression in the BWO group at 24 and 48 h was similar to the expression in mock and control groups, whereas the expression in the BECPs remained significantly low when compared to BWO, mock and control groups (**Supplementary Table S11**).

When compared to control, the mock group showed increased *mmp9* expression at all times, peaking at 6 h post-challenge. In the ECPs group, *mmp9* expression was higher than in controls at 3, 6 and 24 h, but was lower than in mock group at all-time points, reaching a minimum at 48 h post-challenge (**Supplementary Table S10**). Regarding the trial involving injection of bacteria, expression of *mmp9* was lower in the BECPs group, when compared to mock and BWO (**Supplementary Table S11**).

Regarding the antimicrobial peptide hepcidin (*hamp1*), a significant increase in its expression was seen at all sampling time points for the ECPs group, when compared to mock or control groups (**Figure 4F**). This suggests that this iron withholding mechanism may have a preponderant role in the initial response against *T. maritimum*. The highest expression levels of *hamp1* were reached at 3 and 6 h post-challenge, with a 25 and 7-fold increase when compared to the mock group (**Figure 5F**). Moreover, significant upregulation was also seen at all sampling time points for the BECPs group when compared with the control and mock-challenged fish (**Figure 5F**). At 3 and 6 h post-challenge, the group i.p. injected with BECPs group showed approximately a 137- and 36-fold increase in *hamp1* expression, respectively, when compared to the mock group (**Figure 5F**).

As expected, an opposite pattern was observed for the iron exporter ferroportin (*fpn*), since the ECPs group remained always with lower expression values than the control and mock-challenged groups (**Figure 4G**). A similar trend was recorded for fish inoculated with bacteria. At 3 and 6 h, *fpn* expression in the BWO group was lower than in mock group, but afterwards (24 and 48 h) returned to levels similar to the mock group (**Figure 5G**). In contrast, expression of *fpn i*n the BECPs group was downregulated, relative to mock, at all time points analysed (**Figure 5G**).

The expression of *mif, mcsfr* and *mhcII* did not show any major differences and no differences were recorded for *casp1* and *hsp70* expression (**Supplementary Tables S10**, **S11**).

## 4 Discussion

One of the factors that can compromise new advancements in the characterization of the complex host-pathogen relationship operating in tenacibaculosis, which is essential for developing effective prevention measures against the disease is, undoubtedly, the establishment of a suitable infection model, able to mimic the natural infection. Despite the efforts made in the last decades to approach the specific traits and mechanisms of *T. maritimum* pathogenesis, no studies were performed to investigate the host’s immune response against *T. maritimum* infection through different inoculation routes.

Many studies have explored several ways to develop challenge models for *T. maritimum* in different commercial fish species which included Atlantic salmon, rainbow trout and European sea bass, among others (18, 24, 53). These studies allowed a better understanding of the clinical symptoms and/or mortality rates induced by the different inoculation routes for *T. maritimum*, but the host’s immune response was not investigated. Moreover, despite the evidence pointing to an important virulence role of the *T. maritimum* ECPs (13, 20, 23), there is a lack of studies addressing the immune response triggered by the ECPs in the host.

Since the ‘90s, several pathogenicity studies were developed with *T. maritimum*, some of which involved the i.p. route as an inoculation method. Studies involving different serotypes and doses reported that, regardless of the serotype or dose used, *T. maritimum* isolates were not able to induce disease when i.p. inoculated in turbot (13). In a more recent study, Faílde et al. (27), demonstrated that the i.p. inoculation of 10^8^ CFU fish^-1^ led to septicaemia in turbot, but cutaneous lesions characteristic of natural *T. maritimum* infections were not observed in the challenged fish. The toxicity of *T. maritimum*’s ECPs was also investigated by Van Gelderen et al. (20) through i.p. administration of ECPs (1000, 500, 250, 125 and 62.5 µg protein fish^-1^) in Atlantic salmon (average weight of 40 g), revealing a LD_50_ of 3.1 μg of protein g^−1^ of fish body weight. This study showed that i.p. injection of ECPs caused haemorrhages and ascites in the peritoneal cavity, and histological examination of organs collected from fish injected with 1 mg ECPs showed focal inflammation and necrosis in the liver (20).

In the present study, a 100% survival was recorded for all i.p. challenged fish, independently of the inocula used, although they displayed darkened skin during the first 24 h. These results are in agreement with the previously mentioned studies from Avendaño-Herrera et al. (13, 26) and Faílde et al. (26, 27), where no mortality was recorded for turbot i.p. injected with *T. maritimum* or *T. maritimum* ECPs. The here reported findings suggest that bath infection is the best approach to induce tenacibaculosis in European sea bass since i.p. injection did not induce mortality or disease symptoms typically observed in fish suffering from natural *T. maritimum* infections.

Although i.p. inoculation of *T. maritimum* was not able to induce disease in European sea bass, viable bacteria were isolated at 3 h post-challenge from blood and peritoneal exudates of fish injected with BWO and BECPs, indicating that the bacteria were able to persist and reach the systemic circulation. At 24 h post-challenge, no bacterial growth was recorded in blood or peritoneal exudates, indicating that *T. maritimum* is cleared by the host between the 6 and 24 h post-challenge. This ability to clear *T. maritimum* can be related to the rapid and orchestrated response of the host’s resident immune cells in the peritoneal cavity and of the immune cells that migrate to the peritoneal cavity after injection. In the peritoneal exudates, a more exacerbated response of neutrophils, macrophages, lymphocytes and thrombocytes was seen for the fish challenged with BECPs, especially at 6 h post-challenge, suggesting that bacteria and ECPs act synergistically and induce stronger chemotactic signals than bacteria alone. Moreover, the observed leukopenia at the beginning of the trial, associated with lymphopenia and thrombocytopenia, is consistent with acute inflammation, which is known to be triggered in fish by pathogens (including Gram-negative pathogenic bacteria) (54, 55). Again, these effects appeared to be more pronounced in fish challenged with BECPs at 3 h post-challenge, suggesting the occurrence of a stronger immune stimulus and chemotactic effect triggered at the peritoneal cavity by that treatment. Also supporting the immune cells recruitment hypothesis is the enhanced expression of the pro-inflammatory biomarker, *il8*, known for its chemoattractant abilities of inflammatory cells and lymphocytes, which participate in the elimination of bacteria (56, 57). Several studies in fish reported the chemotactic effect of recombinant IL-8 towards neutrophils, macrophages, head-kidney leucocytes and peripheral blood lymphocytes (58–61). The rapid clearance of i.p. injected *T. maritimum*, in contrast to the development of progressive disease after bath challenge, supports the possibility that the route of entry of this pathogen is crucial for its pathogenesis. *T. maritimum*’s adhesion and gliding motility capacities, iron uptake systems, type IX secretion system, as well as its ECPs production, have been suggested to be essential for the immune evasion of the host response, invasion, colonization and nutrient scavenging of these bacteria (21). However, *T. maritimum* was not able to proliferate and trigger the disease when inoculated by i.p. injection. It is likely that the fast-acting host response triggered after i.p. inoculation, with the recruitment of neutrophils, macrophages and other immune cells (62, 63), contributes to counteract the immune evasion ability of *T. maritimum*, and consequently to its rapid clearance. This can also explain the lack of significant responses in the evaluated immune and oxidative stress parameters. Nevertheless, the increase in the plasma bactericidal activity at the end of the trial in fish challenged with BWO and BECPs treatments denotes an attempt to prevent bacterial colonization, since the increase of bactericidal activity in fish was already associated with the detection of pathogens by the host’s innate immune system (16, 64). Furthermore, it may indicate that European sea bass plasma contains bactericidal compounds suitable to eliminate *T. maritimum*. The innate mechanisms against bacterial invasion include a plethora of broad-spectrum antibacterial compounds, which include acute phase proteins, cytokines, non-classical complement activation, phagocytosis and inflammation (64, 65). It is reasonable to speculate that in addition to the augmented bactericidal activity in plasma, the influx of phagocytic cells known to produce bactericidal compounds (31, 66) seen in the peritoneal cavity/infection site, may also have contributed to the elimination of *T. maritimum* after i.p. inoculation.

It is common for diseased fish to present a decrease in several haematological parameters, including RBCs, erythrocytes indices and haemoglobin when exposed to bacteria (67). In the present study, the only variations were recorded for the fish challenged with BECPs, with a tendency to decreased RBCs and haematocrit values from 24 h onwards. The lack of changes in the parameters related to humoral and cellular innate responses, as well as in the oxidative stress indicators, denotes a lack of systemic response to all treatments, which can indicate that regardless of the inoculum type, *T. maritimum* was quickly eliminated by the host’s immune system. Despite the lack of studies approaching *T. maritimum*’s ECPs immunogenic capacity, Salati et al. (19) used formalin-killed cells, crude lipopolysaccharides and ECPs preparations obtained from *T. maritimum* (strain SPVId) as experimental vaccines against tenacibaculosis. After i.p. injection into European sea bass, all preparations, including the ECPs, triggered an immune response, inducing an increase in agglutinating antibody titer and *in vitro* phagocytosis by total blood leukocytes (19). In the present study, although displaying a damper chemotactic effect regarding the peritoneal cavity cells, the treatment with ECPs resulted in a pro-inflammatory response in the head-kidney as strong as the BECPs treatment. The profile and kinetics of the expression of pro-inflammatory cytokines revealed a marked up-regulation at short times (few hours) after i.p. inoculation of ECPs or *T. maritimum* with or without ECPs, which is congruent with the occurrence of an acute inflammatory process. Usually, acute inflammation is described to be enough to overcome an infectious challenge (68). In this process, the activated cells release pro-inflammatory cytokines, such as IL1β and tumour necrosis factor-alpha, and chemokines, like IL8 (68). As previously mentioned, this cocktail of cytokines ultimately culminates in the migration of neutrophils, macrophages and lymphocytes to the inflammation site, for infection clearance (69). In the present study, a fast increase in the expression of pro-inflammatory cytokine-related genes (*il1β*, *tnfα*, *il6*, *il8*) and of *hamp1* gene was detected after injection of BWO, BECPs and ECPs, with the strongest increase registered in the BECPs and ECPs treatments. This type of response is often triggered against bacterial pathogens (70–72). Moreover, interleukin 10 was also overexpressed in those same treatments with a slight delay regarding the expression of the other cytokines, which is consistent with its role in the control and resolution of inflammation (73). The downregulation of ferroportin (an iron exporter) seen in the concentrated ECPs and BECPs treatments throughout the trial is likely triggered by the increased hepcidin as a strategy to prevent iron from being accessible for bacterial growth and constrain bacterial invasion (74). Hepcidin can bind to ferroportin forming a complex that is internalized and degraded, allowing the iron to be retained in the erythrocytes (74, 75). The comparison between BWO and BECPs treatments regarding the studied molecular markers suggests that fish challenged only with bacteria require more time to assemble an innate immune response, which is consistent with a slightly subtler immunogenic effect. *T. maritimum* is a proteolytic pathogen (76), relying on the secretion of ECPs, which include caseinases, gelatinases, amylases and hemolysins (20, 23), to successfully invade and colonize the host’s tissues. The results of the present study suggest that this proteolytic cocktail of ECPs (also demonstrated by the ECPs identification and analysis in the present study), shapes the interaction of *T. maritimum* with its host, corroborating the role of ECPs as the main factors in *T. maritimum*’s pathogenicity, even after inoculation by a route different from its natural route of entry.

Due to its widespread geographical distribution and ubiquitous host species, tenacibaculosis outbreaks have been rising in the last few years, with serious consequences for the aquaculture industry, namely the global salmonid aquaculture industry (11). Despite its current importance as a bacterial pathogen, there is still a lot to explore regarding the complex relationship between *T. maritimum* and its hosts. Although it is not a challenge model that mimics *T. maritimum*’s natural conditions to develop pathogenesis, the i.p. challenge provided a different insight regarding this pathogen’s vulnerability when in contact with the fast and orchestrated host’s innate immune response. The insipid host’s systemic immune response supports the hypothesis of a triggered local acute inflammatory process, which rapidly controls *T. maritimum*’s invasion. The combination of bacteria and its ECPs triggered the most enhanced inflammatory response, although *T. maritimum*’s ECPs were also able to stimulate a similar response, as demonstrated by the pro-inflammatory molecular biomarkers. Undoubtedly, the route of entry of *T. maritimum* greatly influences the immune response triggered in the host and is a determinant factor for a successful host invasion and colonisation.

## Data Availability

The datasets presented in this study can be found in online repositories. The names of the repository/repositories and accession number(s) can be found in the article/**Supplementary Material**.

## References

[1] Shefat SHT. Vaccines for infectious bacterial and viral diseases of fish. J Bacteriology Infect Dis. (2018) 2:1–5. https://www.alliedacademies.org/articles/vaccines-for-infectious-bacterial-and-viral-diseases-of-fish-11056.html.

[2] Aich N Ahmed N Paul A. Issues of antibiotic resistance in aquaculture industry and its way forward. Int J Curr Microbiol Appl Sci. (2018) 7:26–41. doi: 10.20546/IJCMAS.2018.708.004

[3] Bayliss SC Verner-Jeffreys DW Bartie KL Aanensen DM Sheppard SK Adams A. The promise of whole genome pathogen sequencing for the molecular epidemiology of emerging aquaculture pathogens. Front Microbiol. (2017) 8:121. doi: 10.3389/FMICB.2017.00121 28217117 PMC5290457

[4] de Bruijn I Liu Y Wiegertjes GF Raaijmakers JM. Exploring fish microbial communities to mitigate emerging diseases in aquaculture. FEMS Microbiol Ecol. (2018) 94:161. doi: 10.1093/FEMSEC/FIX161 29206925

[5] Moustafa M Eissa A Laila L Gaafar A. Mass Mortalities in Mari-Cultured European Sea Bass (*Dicentrarchus labrax*) at Northern Egypt. Res J Pharmaceutical Biol Chem Sci. (2014) 95–109.

[6] Moustafa M Eissa A Laila A Gaafar A Abumourad I Elgendy M. Investigations into the Potential Causes of Mass Kills in Mari-Cultured Gilthead Sea Bream (*Sparus aurata*) at Northern Egypt. *Research* . J Pharmaceutical Biol Chem Sci. (2015) 6:466–77. https://www.academia.edu/55530972/Investigations_into_the_Potential_Causes_of_Mass_Kills_in_Mari_Cultured_Gilthead_Sea_Bream_Sparus_aurata_at_Northern_Egypt.

[7] Piñeiro-Vidal M Centeno-Sestelo G Riaza A Santos Y. Isolation of pathogenic *Tenacibaculum maritimum*-related organisms from diseased turbot and sole cultured in the Northwest of Spain. Bull Eur Ass Fish Pathol. (2007) 27:29.

[8] Apablaza P Frisch K Brevik Ø.J Småge SB Vallestad C Duesund H. Primary Isolation and Characterization of *Tenacibaculum maritimum* from Chilean Atlantic Salmon Mortalities Associated with a Pseudochattonella spp. Algal Bloom. J Aquat Anim Health. (2017) 29:143–9. doi: 10.1080/08997659.2017.1339643 28613984

[9] Haridy M Hasheim M El-Galil MA Sakai H Yanai T. Pathological findings of *Tenacibaculum maritimus* infection in black damselfish, *Neoglyphieodon melas* and Picasso triggerfish, *Rhinecanthus assasi* in Red Sea, Egypt. J Veterinary Sci Technol. (2014) 6(2). doi: 10.4172/2157-7579.1000214

[10] Lopez P Saulnier D Swarup-Gaucher S David R Lau C Taputuarai R. First Isolation of Virulent *Tenacibaculum maritimum* Isolates from Diseased Orbicular Batfish (*Platax orbicularis*) Farmed in Tahiti Island. Pathogens. (2022) 11:131. doi: 10.3390/PATHOGENS11020131/S1 35215075 PMC8877024

[11] Mabrok M Algammal AM Sivaramasamy E Hetta HF Atwah B Alghamdi S. Tenacibaculosis caused by *Tenacibaculum maritimum*: Updated knowledge of this marine bacterial fish pathogen. Front Cell Infection Microbiol. (2023) 12:1068000. doi: 10.3389/FCIMB.2022.1068000 PMC9853564 36683696

[12] Van Gelderen R Carson J Nowak B. Experimentally induced marine flexibacteriosis in Atlantic salmon smolts *Salmo salar* . Dis Aquat Organisms. (2011) 95:125–35. doi: 10.3354/DAO02329 21848120

[13] Avendaño-Herrera R Toranzo AE Magariños B. Tenacibaculosis infection in marine fish caused by *Tenacibaculum maritimum*: a review. Dis Aquat Organisms. (2006) 71:255–66. doi: 10.3354/DAO071255 17058606

[14] Nowlan JP Lumsden JS Russell S. Advancements in characterizing tenacibaculum infections in Canada. Pathogens. (2020) 9:1029. doi: 10.3390/PATHOGENS9121029 33302445 PMC7763822

[15] Frisch K Smage SB Vallestad C Duesund H Brevik J Klevan A. Experimental induction of mouthrot in Atlantic salmon smolts using *Tenacibaculum maritimum* from Western Canada. J Fish Dis. (2018) 41:1247–58. doi: 10.1111/JFD.12818 29761493

[16] Mabrok M MaChado M Serra CR Afonso A Valente LMP Costas B. Tenacibaculosis induction in the Senegalese sole (*Solea Senegalensis*) and studies of *Tenacibaculum maritimum* survival against host mucus and plasma. J Fish Dis. (2016) 39:1445–55. doi: 10.1111/JFD.12483 27134184

[17] Nishioka T Watanabe KI Sano M. A bath challenge method with *Tenacibaculum maritimum* for Japanese flounder *Paralichthys olivaceus* . Fish Pathol. (2009) 44:178–81. doi: 10.3147/JSFP.44.178

[18] Powell M Carson J Van Gelderen R. Experimental induction of gill disease in Atlantic salmon *Salmo salar* smolts with *Tenacibaculum maritimum* . Dis Aquat Organisms. (2004) 61:179–85. doi: 10.3354/DAO061179 15609873

[19] Salati F Cubadda C Viale I Kusuda R. Immune response of sea bass *Dicentrarchus labrax* to *Tenacibaculum maritimum* antigens. Fisheries Sci. (2005) 3:563–7. doi: 10.1111/J.1444-2906.2005.01000.X

[20] Van Gelderen R Carson J Nowak B. Effect of extracellular products of *Tenacibaculum maritimum* in Atlantic salmon, *Salmo salar* L. J Fish Dis. (2009) 32:727–31. doi: 10.1111/J.1365-2761.2009.01032.X 19531097

[21] Pérez-Pascual D Lunazzi A Magdelenat G Rouy Z Roulet A Lopez-Roques C. The Complete Genome Sequence of the Fish Pathogen *Tenacibaculum maritimum* Provides Insights into Virulence Mechanisms. Front Microbiol. (2017) 8:1542. doi: 10.3389/FMICB.2017.01542 28861057 PMC5561996

[22] Abdelaziz MAM. The host/pathogen interaction during experimental infection of Senegalese sole (*Solea Senegalensis*) by *Tenacibaculum maritimum* . (PhD Thesis). Universidade do Porto (2016). doi: 10.1111/jfd.12483

[23] Escribano MP Balado M Toranzo AE Lemos ML Magariños B. The secretome of the fish pathogen *Tenacibaculum maritimum* includes soluble virulence-related proteins and outer membrane vesicles. Front Cell Infection Microbiol. (2023) 13:1197290/BIBTEX. doi: 10.3389/FCIMB.2023.1197290/BIBTEX PMC10288586 37360528

[24] Bernardet J-F Kerouault B Michel C. Comparative Study on *Flexibacter maritimus* Strains Isolated from Farmed Sea Bass (*Dicentrarchus labrax*) in France. Fish Pathol. (1994) 29:105–11. doi: 10.3147/JSFP.29.105

[25] Baxa DV Kawai K Kusuda R. Experimental Infection of *Flexibacter maritimus* in Black Sea Bream (*Acanthopagrus schlegeli*) Fry. Fish Pathol. (1987) 22:105–9. doi: 10.3147/JSFP.22.105

[26] Avendaño-Herrera R Toranzo AE Magariños B. A challenge model for *Tenacibaculum maritimum* infection in turbot, *Scophthalmus maximus* (L.). J Fish Dis. (2006) 29:371–4. doi: 10.1111/J.1365-2761.2006.00712.X 16768718

[27] Faílde LD Losada AP Bermúdez R Santos Y Quiroga MI. *Tenacibaculum maritimum* infection: Pathology and immunohistochemistry in experimentally challenged turbot (*Psetta maxima* L.). Microbial Pathogenesis. (2013) 65:82–8. doi: 10.1016/J.MICPATH.2013.09.003 24090541

[28] Avendaño-Herrera R Magariños B Moriñigo MA Romalde JL Toranzo AE. A novel O-serotype in *Tenacibaculum maritimum* strains isolated from cultured sole (*Solea Senegalensis*). Bull Eur Ass Fish Pathol. (2005) 25:70.

[29] Ferreira IA Peixoto D Losada AP Quiroga MI Vale A Costas B. Early innate immune responses in European sea bass (*Dicentrarchus labrax* L.) following *Tenacibaculum maritimum* infection. Front Immunol. (2023) 14:1254677. doi: 10.3389/FIMMU.2023.1254677 37731496 PMC10507263

[30] Laemmli UK. Cleavage of structural proteins during the assembly of the head of bacteriophage T4. Nature. (1970) 227:680–5. doi: 10.1038/227680a0 5432063

[31] MaChado M Azeredo R Díaz-Rosales P Afonso A Peres H Oliva-Teles A. Dietary tryptophan and methionine as modulators of European seabass (*Dicentrarchus labrax*) immune status and inflammatory response. Fish Shellfish Immunol. (2015) 42:353–62. doi: 10.1016/j.fsi.2014.11.024 25463296

[32] Afonso A Silva J Lousada S Ellis AE Silva MT. Uptake of neutrophils and neutrophilic components by macrophages in the inflamed peritoneal cavity of rainbow trout (*Oncorhynchus mykiss*). Fish Shellfish Immunol. (1998) 8:319–38. doi: 10.1006/FSIM.1998.0139

[33] Silva MT Silva MN Appelberg R. Neutrophil-macrophage cooperation in the host defence against mycobacterial infections. Microbial Pathogenesis. (1989) 6:369–80. doi: 10.1016/0882-4010(89)90079-X 2770507

[34] Afonso A Ellis AE Silva MT. The leucocyte population of the unstimulated peritoneal cavity of rainbow trout (*Oncorhynchus mykiss*). Fish Shellfish Immunol. (1997) 7:335–48. doi: 10.1006/FSIM.1997.0089

[35] Avendaño-Herrera R Núñez S Magarińos B Toranzo AE. A non-destructive method for rapid detection of *Tenacibaculum maritimum* in farmed fish using nested PCR amplification. Bull Eur Assoc Fish Pathologists. (2004) 24:280–6. https://researchers.unab.cl/en/publications/a-non-destructive-method-for-rapid-detection-of-tenacibaculum-mar.

[36] Toyama T Kita-Tsukamoto K Wakabayashi H. Identification of *Flexibacter maritimus*, *Flavobacterium branchiophilum* and *Cytophaga columnaris* by PCR Targeted 16S Ribosomal DNA. Fish Pathol. (1996) 31:25–31. doi: 10.3147/JSFP.31.25

[37] Osório H Silva C Ferreira M Gullo I Máximo V Barros R. Proteomics analysis of gastric cancer patients with diabetes mellitus. J Clin Med. (2021) 10:1–14. doi: 10.3390/JCM10030407 PMC7866049 33494396

[38] Ellis AE. Serum antiproteases in fish. In: Stolen JS Anderson DP Roberson BS, editors. Techniques in Fish Immunology. SOS Publications, Fair Haven, NJ (1990). W. B. V. M. F. T.

[39] Quade MJ Roth JA. A rapid, direct assay to measure degranulation of bovine neutrophil primary granules. Veterinary Immunol Immunopathology. (1997) 3–4:239–48. https://www.infona.pl//resource/bwmeta1.element.elsevier-83957a12-ccda-3451-9a8a-a7d25e1449dc. 10.1016/s0165-2427(97)00048-2 9436268

[40] Costas B Conceição LEC Dias J Novoa B Figueras A Afonso A. Dietary arginine and repeated handling increase disease resistance and modulate innate immune mechanisms of Senegalese sole (*Solea Senegalensis* Kaup 1858). Fish Shellfish Immunol. (2011) 31:838–47. doi: 10.1016/J.FSI.2011.07.024 21820517

[41] Graham (nee CHUNG) S Jeffries AH Secombes CJ. A novel assay to detect macrophage bactericidal activity in fish: factors influencing the killing of Aeromonas salmonicida. J Fish Dis. (1988) 11:389–96. doi: 10.1111/j.1365-2761.1988.tb00734.x

[42] Saeij JPJ Verburg-Van Kemenade LBM Van Muiswinkel WB Wiegertjes GF. Daily handling stress reduces resistance of carp to *Trypanoplasma borreli: In vitro* modulatory effects of cortisol on leukocyte function and apoptosis. Dev Comp Immunol. (2003) 27:233–45. doi: 10.1016/S0145-305X(02)00093-9 12590974

[43] Bird RP Draper HH. Comparative studies on different methods of malonaldehyde determination. Methods Enzymology. (1984) 105:299–305. doi: 10.1016/S0076-6879(84)05038-2 6727668

[44] Peixoto D Pinto W Gonçalves AT MaChado M Reis B Silva J. Microalgal biomasses have potential as ingredients in microdiets for Senegalese sole (*Solea Senegalensis*) post-larvae. J Appl Phycology. (2021) 33:2241–50. doi: 10.1007/S10811-021-02431-1/FIGURES/2

[45] Claiborne A. Catalase activity. In: Greenwald RA, Editor. CRC Handbook of Methods for Oxygen Radical Research. Boca Raton: CRC Press (1985). p. 283–4. Available at: https://www.scirp.org/(S(351jmbntvnsjt1aadkposzje))/reference/ReferencesPapers.aspx?ReferenceID=796054.

[46] Rodrigues ACM Gravato C Quintaneiro C Bordalo MD Barata C Soares AMVM. Energetic costs and biochemical biomarkers associated with esfenvalerate exposure in *Sericostoma vittatum* . Chemosphere. (2017) 189:445–53. doi: 10.1016/J.CHEMOSPHERE.2017.09.057 28957762

[47] Almeida JR Oliveira C Gravato C Guilhermino L. Linking behavioural alterations with biomarkers responses in the European seabass *Dicentrarchus labrax* L. exposed to the organophosphate pesticide fenitrothion. Ecotoxicology. (2010) 19:1369–81. doi: 10.1007/S10646-010-0523-Y 20686920

[48] Hamre K Penglase SJ Rasinger JD Skjærven KH Olsvik PA. Ontogeny of redox regulation in Atlantic cod (*Gadus morhua*) larvae. Free Radical Biol Med. (2014) 73:337–48. doi: 10.1016/J.FREERADBIOMED.2014.05.017 24873722

[49] Tietze F. Enzymic method for quantitative determination of nanogram amounts of total and oxidized glutathione: Applications to mammalian blood and other tissues. Analytical Biochem. (1969) 27:502–22. doi: 10.1016/0003-2697(69)90064-5 4388022

[50] Kuhl H Beck A Wozniak G Canario AVM Volckaert FAM Reinhardt R. The European sea bass *Dicentrarchus labrax* genome puzzle: Comparative BAC-mapping and low coverage shotgun sequencing. BMC Genomics. (2010) 11:1–13. doi: 10.1186/1471-2164-11-68/FIGURES/5 20105308 PMC2837037

[51] Pfaffl MW. A new mathematical model for relative quantification in real-time RT-PCR. Nucleic Acids Res. (2001) 29:E45. doi: 10.1093/NAR/29.9.E45 11328886 PMC55695

[52] Gorasia DG Veith PD Reynolds EC. The type IX secretion system: advances in structure, function and organisation. Microorganisms. (2020) 8:1–9. doi: 10.3390/MICROORGANISMS8081173 PMC7463736 32752268

[53] Soltani M Munday BL Burke CM. The relative susceptibility of fish to infections by *Flexibacter columnaris* and *Flexibacter maritimus* . Aquaculture. (1996) 140:259–64. doi: 10.1016/0044-8486(95)01157-9

[54] Campbell TW. Exotic Animal Hematology and Cytology, 4th ed. John Wiley & Sons, Inc. (2015). pp. 1–402. doi: 10.1002/9781118993705

[55] Clauss TM Dove ADM Arnold JE. Hematologic disorders of fish. Veterinary Clinics North America: Exotic Anim Pract. (2008) 11:445–62. doi: 10.1016/J.CVEX.2008.03.007 18675728

[56] Brennan K Zheng J. Interleukin 8. XPharm: Compr Pharmacol Reference. (2007), 1–4. doi: 10.1016/B978-008055232-3.61916-6

[57] Havixbeck JJ Barreda DR. Neutrophil development, migration, and function in teleost fish. Biology. (2015) 4:715. doi: 10.3390/BIOLOGY4040715 26561837 PMC4690015

[58] Wang GL Wang MC Zhang XW Chang MX Xie HX Nie P. Molecular cloning, biological effect, and tissue distribution of interleukin-8 protein in mandarin fish (*Siniperca chuasti*) upon *Flavobacterium columnare* infection. Fish Shellfish Immunol. (2017) 66:112–9. doi: 10.1016/J.FSI.2017.05.016 28478260

[59] Wang TT Song XH Bao GM Zhao LX Yu X Zhao J. Molecular characterization, expression analysis, and biological effects of interleukin-8 in grass carp *Ctenopharyngodon idellus* . Fish Shellfish Immunol. (2013) 35:1421–32. doi: 10.1016/J.FSI.2013.08.006 23994423

[60] Wang X Ma G Zhang R Liu L Zhu J Zhu H. Molecular characterization and biological functioning of interleukin-8 in Siberian sturgeon (*Acipenser baeri*). Fish Shellfish Immunol. (2019) 90:91–101. doi: 10.1016/J.FSI.2019.04.010 30978450

[61] Zhao M Liu Y Gao Y Wang X Zhou H Zhang A. Insights into the functional role of grass carp IL-8 in head kidney leukocytes: pro-inflammatory effects and signalling mechanisms. J Fish Biol. (2022) 100:192–202. doi: 10.1111/JFB.14934 34716580

[62] Bruce TJ Brown ML Bruce TJ Brown ML. A review of immune system components, cytokines, and immunostimulants in cultured finfish species. Open J Anim Sci. (2017) 7:267–88. doi: 10.4236/OJAS.2017.73021

[63] Shi X Chi H Sun Y Tang X Xing J Sheng X. The Early Peritoneal Cavity Immune Response to *Vibrio Anguillarum* Infection and to Inactivated Bacterium in Olive Flounder (*Paralichthys olivaceus*). Microorganisms. (2022) 10:2175. doi: 10.3390/MICROORGANISMS10112175 36363767 PMC9693283

[64] Biller-Takahashi JD Takahashi LS Pilarski F Sebastião FA Urbinati EC. Serum bactericidal activity as indicator of innate immunity in pacu *Piaractus mesopotamicus* (Holmberg 1887). Arquivo Brasileiro Medicina Veterinaria e Zootecnia. (2013) 65:1745–51. doi: 10.1590/S0102-09352013000600023

[65] Ellis AE. Innate host defense mechanisms of fish against viruses and bacteria. Dev Comp Immunol. (2001) 25:827–39. doi: 10.1016/S0145-305X(01)00038-6 11602198

[66] do Vale A Afonso A Silva MT. The professional phagocytes of sea bass (*Dicentrarchus labrax* L.): Cytochemical characterisation of neutrophils and macrophages in the normal and inflamed peritoneal cavity. Fish Shellfish Immunol. (2002) 13:183–98. doi: 10.1006/FSIM.2001.0394 12365730

[67] Ahmed I Reshi QM Fazio F. The influence of the endogenous and exogenous factors on hematological parameters in different fish species: a review. Aquaculture Int. (2020) 28:869–99. doi: 10.1007/S10499-019-00501-3

[68] Soliman AM Barreda DR. The acute inflammatory response of teleost fish. Dev Comp Immunol. (2023) 146:104731. doi: 10.1016/J.DCI.2023.104731 37196851

[69] Abdallah F Mijouin L Pichon C. Skin immune landscape: inside and outside the organism. Mediators Inflammation. (2017) 17. doi: 10.1155/2017/5095293 PMC5664322 29180836

[70] Reyes-Cerpa S Maisey K Reyes-López F Toro-Ascuy D Sandino AM Imarai M. Fish Cytokines and Immune Response. In: Turker H, editor. New Advances and Contributions to Fish Biology (2012). doi: 10.5772/53504

[71] Rodrigues P Vázquez-Dorado S Neves JV Wilson JM. Dual function of fish hepcidin: response to experimental iron overload and bacterial infection in sea bass (*Dicentrarchus labrax*). Dev Comp Immunol. (2006) 30:1156–67. doi: 10.1016/J.DCI.2006.02.005 16616368

[72] Shike H Lauth X Westerman ME Ostland VE Carlberg JM Van Olst JC. Bass hepcidin is a novel antimicrobial peptide induced by bacterial challenge. Eur J Biochem. (2002) 269:2232–7. doi: 10.1046/J.1432-1033.2002.02881.X 11985602

[73] Forlenza M Fink IR Raes G Wiegertjes GF. Heterogeneity of macrophage activation in fish. Dev Comp Immunol. (2011) 35:1246–55. doi: 10.1016/J.DCI.2011.03.008 21414343

[74] Ward RJ Crichton RR Taylor DL Della Corte L Srai SK Dexter DT. Iron and the immune system. J Neural Transm (Vienna Austria : 1996). (2011) 118:315–28. doi: 10.1007/S00702-010-0479-3/FIGURES/10 20878427

[75] Nemeth E Tuttle MS Powelson J Vaughn MD Donovan A Ward DMV. Hepcidin regulates cellular iron efflux by binding to ferroportin and inducing its internalization. Science. (2004) 306:2090–3. doi: 10.1126/SCIENCE.1104742/SUPPL_FILE/NEMETH.SOM.REVISED.PDF 15514116

[76] Wakabayashi H Hikida M Masumura K. *Flexibacter maritimus* sp. nov., a Pathogen of Marine Fishes. Int J Systematic Evolutionary Microbiol. (1986) 36:396–8. doi: 10.1099/00207713-36-3-396

