# Supplementary material for: Tenacibaculum maritimum can boost inflammation in Dicentrarchus labrax upon peritoneal injection but cannot trigger tenacibaculosis disease
Source: Front Immunol. 2024 Oct 14;15:1478241. doi: 10.3389/fimmu.2024.1478241 (PMC11513285; doi:10.3389/fimmu.2024.1478241)
Supplement: Supplementary file 1 [file DataSheet1.pdf]

Supplementary materials

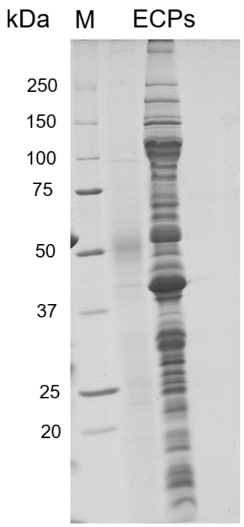

Figure S1: Coomassie-blue stained SDS-PAGE gel of the cell-free concentrated *T. maritimum* ECPs used to challenge European sea bass (*Dicentrarchus labrax*) by intraperitoneal injection (i.p.). The lane contains protein equivalent to 1 mL of concentrated ECPs. M- Molecular weight marker. Numbers on the left indicate the molecular weight of the markers (Precision Plus Protein™ Unstained Protein Standards, Bio-Rad), in kDa.

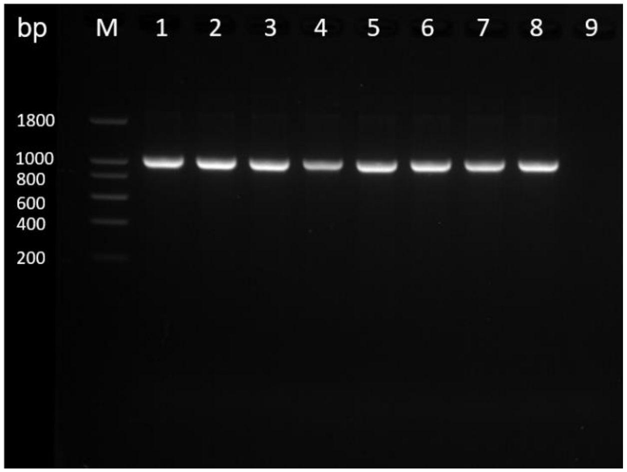

Figure S2: PCR products obtained with MAR1 and MAR2 using bacterial genomic DNA extracted from randomly selected colonies obtained from peritoneal exudates and blood samples of challenged fish (Lanes 1-7); Lane 8- positive control - *T. maritimum* strain ACC13.1; Lane 9- negative control - sterile distilled water); M: NZYDNA Ladder I (NZYTech, Lisbon, Portugal) (200 to 1800 bp). Numbers on the left indicate the size of the marker, in bp.

Table S1: *T. maritimum*'s proteins identified by NanoLC-MS/MS in concentrated ECPs used to challenge *D. labrax* by i.p. injection. The table shows hits with coverage above 30%, unique peptides superior to 3 and SEQUEST HT score greater than 100.

Accession	Name	Gene names	Coverage (%)	Unique Peptides	MW (kDa)	calc. pI	Score Sequest HT	Gene Ontology (GO)	Go Term
Potential virulence factors									
A0A2H1ECB7	Exo-alpha-sialidase	siaA MARIT_2686	55	103	245.5	5.4	2551.5	Carbohydrate metabolic process	GO:0005975
A0A2H1EER9	Probable M43 family metalloprotease containing a C-terminal secretion signal	MARIT_3130	40	15	95.6	5.3	2140.4	Proteolysis	GO:0006508

A0A2H1ECV9	Probable M14 family carboxypeptidase containing a C-terminal secretion signal	MARIT_2507	53	47	147.6	6.6	1914.9	Proteolysis	GO:0006508
A0A2H1EB05	Probable S8 family protease containing a C-terminal secretion signal	MARIT_2055	45	22	59.6	8.6	360.2	Proteolysis	GO:0006508
A0A2H1E5T3	Secreted subtilase family protein, peptidase S8	MARIT_0203	40	20	59.2	6.7	102.6	Proteolysis	GO:0006508
Adhesins									
A0A2H1E8R3	Adhesin SprB	sprB MARIT_1321	35	105	625.5	4.5	1308.0	-	-
A0A2H1E9U3	Adhesin SprC	sprC MARIT_1318	44	28	80.1	4.9	213.8	-	-
Iron metabolism									
A0A2H1E9M2	Iron regulated protein Imelysin family lipoprotein	irpA1 MARIT_1664	60	25	41.2	5.0	482.0	-	-
A0A2H1ECT1	Heme binding lipoprotein HmuY-family	MARIT_2477	60	14	27.2	5.6	218.5	-	-
A0A2H1E921	Heme binding lipoprotein HmuY-family	MARIT_1313	60	17	36.5	6.4	145.6	-	-
Lipoproteins									
A0A2H1EBV5	Probable lipoprotein	MARIT_2470	58	40	46.3	7.9	3747.4	-	-

A0A2H1ED88	Probable lipoprotein	MARIT_3005	67	32	67.8	4.8	2761.2	-	-
A0A2H1E7K5	Probable lipoprotein	MARIT_0907	86	35	48.9	4.9	2115.2	-	-
A0A2H1E9Q6	Probable lipoprotein	MARIT_1705	60	15	44.6	8.1	1327.2	-	-
A0A2H1EDA9	Probable lipoprotein	MARIT_3027	55	18	29.7	5.9	824.8	-	-
A0A2H1E7J0	Probable lipoprotein	MARIT_0452	75	18	32.3	5.2	797.2	-	-
A0A2H1E833	Probable lipoprotein	MARIT_1058	57	15	30.7	5.1	634.4	-	-
A0A2H1E743	Probable lipoprotein	MARIT_0551	55	13	21.8	8.7	253.2	-	-
A0A2H1EA50	Probable lipoprotein	MARIT_1731	37	12	39.6	5.0	193.5	-	-
A0A2H1E5I3	Lipoprotein	MARIT_0099	61	19	32.0	5.0	191.4	-	-
A0A2H1E6T8	Probable lipoprotein	MARIT_0183	59	20	47.7	6.4	184.6	-	-
A0A2H1E6P1	Flagellar motor/Chemotaxis (MotB)-related lipoprotein	MARIT_0403	59	15	31.3	9.4	105.2	Membrane	GO:0016020

Oxidative stress metabolism									
A0A2H1EB32	Superoxide dismutase 2	sodC MARIT_1821	40	6	18.0	6.9	533.4	Copper ion binding; Superoxide dismutase activity	GO:0005507; GO:0004784
A0A2H1EDI6	Superoxide dismutase	sodA MARIT_3105	56	7	22.4	5.3	262.5	Metal ion binding; Superoxide dismutase activity	GO:0046872; GO:0004784
A0A2H1EAC5	Metallo-dependent phosphatase containing a C-terminal secretion signal	MARIT_1816	33	22	102.3	5.2	212.4	Hydrolase activity	GO:0016787
A0A2H1EC90	Thioredoxin	trxA MARIT_2619	79	7	11.5	4.9	208.2	Protein-disulfide reductase activity	GO:0015035
A0A2H1E827	Alkyl hydroperoxide reductase C (Peroxiredoxin) (Thioredoxin peroxidase)	MARIT_0947	72	11	23.6	4.8	117.9	Peroxiredoxin activity	GO:0051920
Outer membrane proteins and TonB-related proteins									
A0A2H1EBZ4	SusC/RagA family TonB-dependent receptor	MARIT_2376	47	47	113.5	5.6	459.5	Cell outer membrane	GO:0009279
A0A2H1ED77	OmpA family protein	MARIT_2995	57	23	48.7	5.1	329.7	Cell adhesion; monoatomic ion transport	GO:0007155; GO:0006811
A0A2H1E6S9	Putative outer membrane protein	MARIT_0582	68	27	53.5	6.1	308.8	-	-
A0A2H1E9H4	Outer membrane protein beta-barrel domain-containing protein	MARIT_1482	31	4	17.1	8.1	189.0	-	-
A0A2H1E9P7	Outer membrane protein beta-barrel domain-containing protein	MARIT_1564	56	13	22.4	9.0	181.3	-	-

A0A2H1E5Y7	TonB-dependent outer membrane receptor	MARIT_0270	40	28	103.0	8.5	173.0	Cell outer membrane	GO:0009279
A0A2H1E6W5	TonB-dependent outer membrane receptor	MARIT_0214	47	33	102.7	6.5	168.7	Cell outer membrane	GO:0009280
A0A2H1EAX6	TonB-dependent outer membrane receptor	MARIT_1756	36	33	116.1	5.5	168.4	Cell outer membrane	GO:0009281
A0A2H1E9Y1	TonB-dependent outer membrane receptor	MARIT_1457	45	30	100.0	5.4	155.2	Cell outer membrane	GO:0009282
A0A2H1E930	TonB-dependent outer membrane receptor	MARIT_1458	34	25	101.0	6.4	152.3	Cell outer membrane	GO:0009283
A0A2H1EDN0	TonB-dependent outer membrane receptor	MARIT_3004	39	32	104.5	8.8	127.3	Cell outer membrane	GO:0009284
A0A2H1E607	TonB-dependent outer membrane receptor	MARIT_0268	34	27	104.6	6.2	122.8	Cell outer membrane	GO:0009285
A0A2H1E6Q0	Outer membrane protein beta-barrel domain-containing protein	MARIT_0557	44	7	21.9	9.3	112.2	-	-
A0A2H1E9V9	Outer membrane protein beta-barrel domain-containing protein	MARIT_1624	60	12	30.7	7.2	109.2	-	-
Secretion system-related proteins									
A0A2H1E9D1	Secretion system C-terminal sorting domain-containing protein	MARIT_1397	43	8	37.0	5.5	222.9	-	-

A0A2H1E7X7	Gliding motility lipoprotein GldJ	gldJ MARIT_0896	35	22	65.3	9.3	162.6	-	-
A0A2H1E7N1	Gliding motility lipoprotein GldK	gldK MARIT_0754	36	16	52.8	6.5	146.9	-	-
A0A2H1E7A0	Gliding motility protein GldN	gldN MARIT_0757	61	18	33.5	5.3	125.3	-	-
A0A2H1E7X8	Por secretion system protein porV	porV MARIT_0894	55	14	40.0	5.2	119.8	-	-

Table S2: Haematological parameters of European sea bass i.p. challenged with MB (Mock) or *T. maritimum*'s ECPs (ECPs). Data are expressed as mean ± SEM (n=12 *per* treatment). Different lowercase letters stand for significant differences between treatments among time points and different symbols represent significant differences between the control group (undisturbed) and the remaining groups (*Student's* t-test; $p \leq 0.05$). WBC - white blood cells; RBC - red blood cells; Ht - haematocrit; Hg - haemoglobin; MCV - mean corpuscular volume; MCH - mean corpuscular haemoglobin; MCHC - mean corpuscular haemoglobin concentration.

Parameters	Control	Mock-challenged				ECPs			
	**0 h**	**3 h**	**6 h**	**24 h**	**48 h**	**3 h**	**6 h**	**24 h**	**48 h**
**WBC (x10^4/µL)**	5.10±0.19**#**	4.25±0.30	1.16±0.19*****	1.89±0.17*****	1.26±0.12*****	3.48±0.30*****	1.28±0.20*****	1.75±0.17*****	1.24±0.17*
**RBC (x10^6/µL)**	2.28±0.11**#**	2.69±0.10*****	2.49±0.09	2.74±0.12*****	2.30±0.13	2.47±0.07	2.26±0.09	2.73±0.12	2.24±0.13
**Ht (%)**	25.64±1.22**#**	28.17±0.58	29.00±1.17	25.50±1.38*****	31.00±1.39**a**	28.58±0.70	26.27±1.14	27.08±1.38	26.58±0.74**b**
**Hg (g/dL)**	2.66±0.14	2.65±0.05	2.82±0.12	2.75±0.12	2.87±0.12	2.65±0.06	2.58±0.04	2.99±0.12	2.76±0.09
**MCV (µm^3)**	113.53±5.33**#**	106.32±4.18	117.90±5.74	93.32±4.06*****	137.57±6.51*****	116.79±4.54	116.74±4.90	104.23±4.06	124.27±8.81
**MCH (pg/cell)**	11.80±0.60**#**	10.00±0.41*****	11.54±0.69	10.15±0.45*****	12.76±0.57	10.84±0.42	11.62±0.45	11.70±0.45	13.05±1.15
**MCHC (g/100 mL)**	10.93±0.40**#**	9.42±0.21*****	9.27±0.33*****	10.35±0.30	9.35±0.35*****	9.33±0.26*****	10.00±0.37	11.13±0.28	10.45±0.40

***Student's* t-test (*p*-values)**

Parameters	Mock-challenged x ECPs			
	**3 h**	**6 h**	**24 h**	**48 h**

**WBC (x10⁴/µL)**	0.097	0.523	0.429	0.625
**RBC (x10⁶/µL)**	0.085	0.130	0.537	0.789
**Ht (%)**	0.650	0.110	0.347	0.010
**Hg (g/dL)**	0.935	0.056	0.123	0.458
**MCV (µm³)**	0.104	0.881	0.221	0.237
**MCH (pg/cell)**	0.166	0.925	0.229	0.824
**MCHC (g/100 mL)**	0.782	0.169	0.154	0.050

Table S3: Absolute values (x 10^4/µL) of peripheral blood leukocytes (neutrophils, monocytes, lymphocytes and thrombocytes) of European sea bass i.p. challenged with MB (Mock) or *T. maritimum*'s ECPs (ECPs). Data are expressed as mean ± SEM (n=12 *per* treatment). Different lowercase letters stand for significant differences between treatments among time points and different symbols represent significant differences between the control group (undisturbed) and the remaining groups (*Student's* t-test; $p \leq 0.05$).

Parameters	Control	Mock-challenged				ECPs			
	**0 h**	**3 h**	**6 h**	**24 h**	**48 h**	**3 h**	**6 h**	**24 h**	**48 h**
**Neutrophils ($x10^4$/µL)**	0.27±0.06#	0.15±0.03b	0.08±0.01b*	0.22±0.04	0.05±0.01b*	0.39±0.04a*	0.19±0.03a	0.27±0.02	0.12±0.03a
**Monocytes ($x10^4$/µL)**	0.11±0.01#	0.05±0.01*	0.04±0.01a*	0.06±0.01	0.03±0.00	0.06±0.01	0.01±0.00b*	0.05±0.02*	0.04±0.01
**Lymphocytes ($x10^4$/µL)**	1.34±0.08#	0.83±0.08*	0.23±0.01*	0.49±0.07*	0.26±0.04*	0.64±0.08*	0.20±0.03*	0.37±0.05*	0.29±0.04*
**Thrombocytes ($x10^4$/µL)**	3.39±0.14#	3.21±0.27	0.80±0.14*	1.12±0.14*	0.93±0.09*	2.39±0.20*	0.87±0.16*	1.06±0.15*	0.80±0.11*

***Student's* t-test (*p*-values)**

Parameters	Mock-challenged x ECPs			
	**3 h**	**6 h**	**24 h**	**48 h**
**Neutrophils ($x10^4$/µL)**	<.001	0.004	0.232	0.011
**Monocytes ($x10^4$/µL)**	0.557	<.001	0.657	0.488
**Lymphocytes ($x10^4$/µL)**	0.055	0.631	0.118	0.569
**Thrombocytes ($x10^4$/µL)**	0.060	0.556	0.792	0.217

Table S4: Haematological parameters of European sea bass i.p. challenged with MB (Mock) or 5.5 x 10^5 CFU *T. maritimum* without ECPs (BWO) or 5.5 x 10^5 CFU *T. maritimum* with ECPs (BECPs). Data are expressed as mean ± SEM (n=12 *per* treatment). Different lowercase letters stand for significant differences in treatments among each time point, while different capital letters indicate differences in time among the same treatment (Two-Way ANOVA for interaction between factors, followed by Tukey's HSD or LSD for

multiple comparisons, *p-value* ≤ 0.05). Different symbols represent significant differences between the control group (undisturbed) and the different treatment groups (*Student's* t-test; *p* ≤ 0.05). WBC - white blood cells; RBC - red blood cells; Ht - haematocrit; Hg - haemoglobin; MCV - mean corpuscular volume; MCH - mean corpuscular haemoglobin; MCHC - mean corpuscular haemoglobin concentration.

Parameters	Control	Mock-challenged				BWO				BECPs			
	0 h	3 h	6 h	24 h	48 h	3 h	6 h	24 h	48 h	3 h	6 h	24 h	48 h
**WBC (x10^4/µL)**	5.10±0.19	4.25±0.30	1.16±0.19	1.89±0.17	1.26±0.12	2.17±0.30	1.57±0.15	2.58±0.30	1.31±0.12	1.41±0.21	0.97±0.11	1.73±0.17	1.34±0.17
**RBC (x10^6/µL)**	2.28±0.11	2.69±0.10	2.49±0.09	2.74±0.12	2.30±0.13	2.50±0.14	2.76±0.17	2.78±0.14	2.45±0.20	2.13±0.10	2.20±0.13	2.52±0.18	2.12±0.10
**Ht (%)**	25.64±1.22	28.17±0.58	29.00±1.17	25.50±1.38	31.00±1.39	27.17±1.02	27.36±1.09	25.92±1.33	28.75±1.23	26.11±1.10	23.17±0.96	3.07±0.98	23.67±1.18
**Hg (g/dL)**	2.66±0.14	2.65±0.05	2.82±0.12	2.75±0.12	2.87±0.12	2.50±0.04	2.69±0.13	3.06±0.15	2.94±0.11	2.56±0.04	2.51±0.06	3.07±0.16	2.84±0.08
**MCV (µm^3)**	113.53±5.33	106.32±4.18	117.90±5.74	93.32±4.06	137.57±6.51	111.49±5.26	103.40±4.40	97.66±8.76	109.54±7.75	122.13±11.31	109.10±8.30	92.91±6.55	114.87±8.19
**MCH (pg/cell)**	11.80±0.60	10.00±0.41	11.54±0.69	10.15±0.45	12.76±0.57	10.40±0.72	10.11±0.68	11.28±0.78	13.23±1.65	11.79±0.86	11.83±0.74	11.98±0.57	13.76±0.71
**MCHC (g/100 mL)**	10.93±0.40	9.42±0.21	9.27±0.33	10.35±0.30	9.35±0.35	9.34±0.40	9.97±0.62	12.35±1.13	10.44±0.59	9.93±0.30	10.98±0.36	13.89±0.67	12.37±0.71

One-Way ANOVA

Parameters	Time				Treatment		
	3 h	6 h	24 h	48 h	Mock	BWO	BECPs
**WBC (x10^4/µL)**	-	-	-	-	-	-	-
**RBC (x10^6/µL)**	AB	AB	A	B	a	a	b
**Ht (%)**	AB	AB	A	B	a	a	b
**Hg (g/dL)**	A	AB	C	BC	-	-	-
**MCV (µm^3)**	A	A	B	A	-	-	-
**MCH (pg/cell)**	A	A	A	B	-	-	-
**MCHC (g/100 mL)**	A	A	B	A	a	a	b

2-Way ANOVA

Parameters	Time x Treatment											
	Mock-challenged				BWO				BECPs			
	3 h	6 h	24 h	48 h	3 h	6 h	24 h	48 h	3 h	6 h	24 h	48 h
**WBC (x10^4/µL)**	Aa	Bb	C	B	Ab	Ba	A	B	ABc	Ab	B	AB

**RBC (x10^6/µL)**	-	-	-	-	-	-	-	-	-	-	-	-
**Ht (%)**	-	-	-	-	-	-	-	-	-	-	-	-
**Hg (g/dL)**	-	-	-	-	-	-	-	-	-	-	-	-
**MCV (µm^3)**	-	-	-	-	-	-	-	-	-	-	-	-
**MCH (pg/cell)**	-	-	-	-	-	-	-	-	-	-	-	-
**MCHC (g/100 mL)**	-	-	-	-	-	-	-	-	-	-	-	-

2-Way ANOVA (*p*-values)

Parameters	**Time**	**Treatment**	**Time x Treatment**
**WBC (x10^4/µL)**	<0.001	<0.001	<0.001
**RBC (x10^6/µL)**	0.006	<0.001	0.671
**Ht (%)**	0.004	<0.001	0.290
**Hg (g/dL)**	<0.001	0.801	0.086
**MCV (µm^3)**	<0.001	0.239	0.081
**MCH (pg/cell)**	0.001	0.059	0.759
**MCHC (g/100 mL)**	<0.001	<0.001	0.475

***Student's* t-test**

Parameters	**Control**	**Mock-challenged**				**BWO**				**BECPs**			
	**0 h**	**3 h**	**6 h**	**24 h**	**48 h**	**3 h**	**6 h**	**24 h**	**48 h**	**3 h**	**6 h**	**24 h**	**48 h**
**WBC (x10^4/µL)**	#	-	*	*	*	*	*	*	*	*	*	*	*
**RBC (x10^6/µL)**	#	*	-	*	-	-	-	*	-	-	-	*	-
**Ht (%)**	#	-	-	-	*	-	-	-	-	-	-	*	-
**Hg (g/dL)**	-	-	-	-	-	-	-	-	-	-	-	-	-
**MCV (µm^3)**	#	-	-	*	*	-	-	-	-	-	-	*	-
**MCH (pg/cell)**	#	*	-	*	-	-	-	-	-	-	-	-	*
**MCHC (g/100 mL)**	#	*	-	-	*	*	-	-	-	-	-	*	-

Table S5: Absolute values (x 10^4/µL) of peripheral blood leukocytes (neutrophils, monocytes, lymphocytes and thrombocytes) of European sea bass i.p. challenged with MB (Mock) or 5.5 x 10^5 CFU *T. maritimum* without ECPs (BWO) or 5.5 x 10^5 CFU *T. maritimum* with ECPs (BECPs). Data are expressed as mean ± SEM (n=12 *per* treatment). Different lowercase letters stand for significant differences in treatments among each time point, while different capital letters indicate differences in time among the same treatment (Two-

Way ANOVA for interaction between factors, followed by Tukey's HSD or LSD for multiple comparisons, *p-value* ≤ 0.05). Different symbols represent significant differences between the control group (undisturbed) and the different treatment groups (*Student's* t-test; p ≤ 0.05).

Parameters	Control	Mock-challenged				BWO				BECPs			
	**0 h**	**3 h**	**6 h**	**24 h**	**48 h**	**3 h**	**6 h**	**24 h**	**48 h**	**3 h**	**6 h**	**24 h**	**48 h**
**Neutrophils (x10^4/µL)**	0.27±0.06	0.15±0.03	0.08±0.01	0.22±0.04	0.05±0.01	0.19±0.03	0.33±0.04	0.34±0.07	0.05±0.01	0.12±0.02	0.12±0.02	0.23±0.03	0.05±0.02
**Monocytes (x10^4/µL)**	0.11±0.01	0.05±0.01	0.04±0.01	0.06±0.01	0.03±0.00	0.06±0.01	0.02±0.01	0.09±0.02	0.03±0.00	0.02±0.01	0.01±0.00	0.05±0.01	0.04±0.01
**Lymphocytes (x10^4/µL)**	1.34±0.08	0.83±0.08	0.23±0.01	0.49±0.07	0.26±0.04	0.33±0.04	0.23±0.03	0.54±0.07	0.36±0.06	0.21±0.03	0.15±0.02	0.30±0.05	0.31±0.05
**Thrombocytes (x10^4/µL)**	3.39±0.14	3.21±0.27	0.80±0.14	1.12±0.14	0.93±0.09	1.58±0.24	0.98±0.10	1.62±0.19	0.87±0.07	1.06±0.16	0.69±0.08	1.15±0.11	0.78±0.13

2-Way ANOVA

Parameters	Time x Treatment											
	Mock-challenged				BWO				BECPs			
	**3 h**	**6 h**	**24 h**	**48 h**	**3 h**	**6 h**	**24 h**	**48 h**	**3 h**	**6 h**	**24 h**	**48 h**
**Neutrophils (x10^4/µL)**	AB	ACb	B	C	A	aB	AB	C	A	Ab	B	C
**Monocytes (x10^4/µL)**	ABa	ABa	A	B	Aa	Bab	A	B	Ab	Ab	B	B
**Lymphocytes (x10^4/µL)**	Aa	Bab	Ca	B	Ab	Aa	Ba	AB	ABc	Ab	Bb	B
**Thrombocytes (x10^4/µL)**	Aa	Bab	C	BC	ABb	BCa	A	C	ACb	Bb	C	AB

2-Way ANOVA (*p*-values)

Parameters	Time	Treatment	Time x Treatment
**Neutrophils (x10^4/µL)**	<0.001	<0.001	0.008
**Monocytes (x10^4/µL)**	<0.001	0.003	0.002
**Lymphocytes (x10^4/µL)**	<0.001	<0.001	<0.001
**Thrombocytes (x10^4/µL)**	<0.001	0.002	<0.001

***Student's* t-test**

Parameters	Control	Mock-challenged				BWO				BECPs			
	**0 h**	**3 h**	**6 h**	**24 h**	**48 h**	**3 h**	**6 h**	**24 h**	**48 h**	**3 h**	**6 h**	**24 h**	**48 h**
**Neutrophils (x10^4/µL)**	#	-	*	-	*	-	-	-	*	*	*	-	*

**Monocytes ($x10^4$/µL)**	#	*	*	-	*	-	*	-	*	*	*	*	*
**Lymphocytes ($x10^4$/µL)**	#	*	*	-	*	*	*	-	*	*	*	-	*
**Thrombocytes ($x10^4$/µL)**	#	-	*	*	*	*	*	*	*	*	*	*	*

Table S6: Immune parameters (antiprotease (%) and proteases activities (%), peroxidase (units/mL), lysozyme (units/mL), bactericidal activity (%) and nitrite concentration (µM)) of plasma of European sea bass i.p. challenged with MB (Mock) or *T. maritimum*'s ECPs (ECPs). Data are expressed as mean ± SEM (n=12 *per* treatment). Different lowercase letters stand for significant differences between treatments among time points and different symbols represent significant differences between the control group (undisturbed) and the remaining groups (*Student's* t-test; p ≤ 0.05). NO - nitrite

Parameters	**Control**	**Mock-challenged**				**ECPs**			
	**0 h**	**3 h**	**6 h**	**24 h**	**48 h**	**3 h**	**6 h**	**24 h**	**48 h**
**Lysozyme (µg/mL)**	10.53±0.72**#**	7.46±0.57*****	8.26±0.70*****	7.97±1.31	6.81±0.96*****	6.28±0.59*****	8.02±0.77*****	6.99±0.35*****	4.93±0.76*****
**Antiprotease activity (%)**	97.83±0.36	98.21±0.20	97.28±0.26	97.34±0.24**b**	97.23±0.24	98.16±0.24	97.40±0.26	98.14±0.17**a**	96.68±0.27*****
**Protease activity (%)**	8.71±0.23	8.68±0.19**b**	8.76±0.23**b**	8.33±0.20	8.15±0.17	9.84±0.32**a**	9.80±0.28**a**	8.06±0.19*****	7.89±0.12*****
**Peroxidase activity (U/mL)**	38.92±6.62	27.23±2.15	30.91±3.19	72.33±7.82*****	61.70±7.77	23.85±3.32	29.87±2.06	61.71±6.14*****	82.31±11.94*****
**Bactericidal activity (%)**	35.84±6.08**#**	45.54±5.48	49.19±3.45	15.67±4.23**b***	27.05±2.25	54.86±2.32*****	50.72±3.61*****	45.22±4.91**a**	23.67±2.45
**NO (µM)**	0.54±0.06	0.41±0.03**b**	0.52±0.07	0.57±0.10	0.48±0.04**b**	0.63±0.07**a**	0.53±0.04	0.57±0.04	0.68±0.05**a**

***Student's* t-test (*p*-values)**

Parameters	**Mock-challenged x ECPs**			
	**3 h**	**6 h**	**24 h**	**48 h**
**Lysozyme (µg/mL)**	0.166	0.819	0.460	0.140
**Antiprotease activity (%)**	0.874	0.746	0.015	0.135
**Protease activity (%)**	0.005	0.009	0.163	0.217
**Peroxidase activity (U/mL)**	0.412	0.787	0.533	0.247
**Bactericidal activity (%)**	0.121	0.763	<.001	0.330
**NO (µM)**	0.011	0.896	0.997	0.022

Table S7: Immune parameters (antiprotease (%) and proteases activities (%), peroxidase (units/mL), lysozyme (units/mL), bactericidal activity (%) and nitrite concentration (µM)) of plasma of European sea bass i.p. challenged with MB (Mock) or 5.5 x 10^5 CFU *T. maritimum* without ECPs (BWO) or 5.5 x 10^5 CFU *T. maritimum* with ECPs (BECPs). Different

lowercase letters stand for significant differences in treatments among each time point, while different capital letters indicate differences in time among the same treatment (Two-Way ANOVA for interaction between factors, followed by Tukey's HSD or LSD for multiple comparisons, *p-value* ≤ 0.05). Different symbols represent significant differences between the control group (undisturbed) and the different treatment groups (*Student's* t-test; $p \leq 0.05$). NO - nitrite

Parameters	**Control**	**Mock-challenged**				**BWO**				**BECPs**			
	**0 h**	**3 h**	**6 h**	**24 h**	**48 h**	**3 h**	**6 h**	**24 h**	**48 h**	**3 h**	**6 h**	**24 h**	**48 h**
**Lysozyme (µg/mL)**	10.53±0.72	7.46±0.57	8.26±0.70	7.97±1.31	6.81±0.96	6.74±0.61	7.70±0.38	6.33±0.83	4.97±0.47	6.38±0.74	7.04±0.58	6.94±0.91	6.16±0.87
**Antiprotease activity (%)**	97.83±0.36	98.21±0.20	97.28±0.26	97.34±0.24	97.23±0.24	97.41±0.34	96.74±0.26	97.36±0.27	97.65±0.17	97.61±0.17	96.88±0.39	97.16±0.27	97.41±0.23
**Protease activity (%)**	8.71±0.23	8.68±0.19	8.76±0.23	8.62±0.34	8.15±0.17	8.61±0.17	9.30±0.34	8.51±0.16	8.64±0.39	9.23±0.39	8.51±0.16	79.49±0.18	7.90±0.28
**Peroxidase activity (U/mL)**	38.92±6.62	27.23±2.15	30.91±3.19	72.33±7.82	61.70±7.77	24.22±1.99	23.96±2.35	76.22±14.72	86.70±15.44	27.86±2.68	33.76±8.83	79.49±14.83	98.54±13.90
**Bactericidal activity (%)**	35.84±6.08	45.54±5.48	49.19±3.45	15.67±4.23	27.05±2.25	49.71±2.68	53.47±1.77	35.96±4.77	45.90±4.89	47.10±2.99	48.99±1.51	31.88±0.90	52.59±4.24
**NO (µM)**	0.54±0.06	0.41±0.03	0.52±0.07	0.57±0.10	0.48±0.04	0.50±0.10	0.56±0.07	0.46±0.08	0.74±0.09	0.51±0.06	0.56±0.05	0.73±0.07	0.60±0.09

One-Way ANOVA

Parameters	**Time**				**Treatment**		
	**3 h**	**6 h**	**24 h**	**48 h**	**Mock**	**BWO**	**BECPs**
**Lysozyme (µg/mL)**	-	-	-	-	-	-	-
**Antiprotease activity (%)**	A	B	AB	AB	-	-	-
**Protease activity (%)**	AB	A	BC	C	-	-	-
**Peroxidase activity (U/mL)**	A	A	B	B	-	-	-
**Bactericidal activity (%)**	-	-	-	-	-	-	-
**NO (µM)**	-	-	-	-	-	-	-

2-Way ANOVA

	**Time x Treatment**											
Parameters	**Mock-challenged**				**BWO**				**BECPs**			
	**3 h**	**6 h**	**24 h**	**48 h**	**3 h**	**6 h**	**24 h**	**48 h**	**3 h**	**6 h**	**24 h**	**48 h**
**Lysozyme (µg/mL)**	-	-	-	-	-	-	-	-	-	-	-	-
**Antiprotease activity (%)**	-	-	-	-	-	-	-	-	-	-	-	-
**Protease activity (%)**	-	-	-	-	-	-	-	-	-	-	-	-

**Peroxidase activity (U/mL)**	-	-	-	-	-	-	-	-	-	-	-	-
**Bactericidal activity (%)**	A	A	Bb	Cb	A	A	Ba	ABa	A	A	Ba	Aa
**NO (µM)**	-	-	-	-	-	-	-	-	-	-	-	-

2-Way ANOVA (*p*-values)

Parameters	Time	Treatment	Time x Treatment
**Lysozyme (µg/mL)**	0.059	0.072	0.911
**Antiprotease activity (%)**	0.004	0.403	0.249
**Protease activity (%)**	0.002	0.118	0.114
**Peroxidase activity (U/mL)**	<.001	0.610	0.714
**Bactericidal activity (%)**	<.001	<.001	0.004
**NO (µM)**	0.057	0.160	0.134

***Student's* t-test**

Parameters	Control	Mock-challenged				BWO				BECPs			
	0 h	3 h	6 h	24 h	48 h	3 h	6 h	24 h	48 h	3 h	6 h	24 h	48 h
**Lysozyme (µg/mL)**	#	*	*	-	*	*	*	*	*	*	*	*	*
**Antiprotease activity (%)**	#	-	-	-	-	-	*	-	-	-	-	-	-
**Protease activity (%)**	#	-	-	-	-	-	-	-	-	-	-	*	*
**Peroxidase activity (U/mL)**	#	-	-	*	*	-	*	*	*	-	-	*	*
**Bactericidal activity (%)**	#	-	-	*	-	-	*	-	-	-	-	-	*
**NO (µM)**	#	-	-	-	-	-	-	-	-	-	-	*	-

Table S8: Oxidative stress biomarkers activity of the liver of European sea bass i.p. challenged with MB (Mock) or *T. maritimum*'s ECPs (ECPs). Data are expressed as mean ± SEM (n=12 *per* treatment). Different lowercase letters stand for significant differences between treatments among time points and different symbols represent significant differences between the control group (undisturbed) and the remaining groups (*Student's* t-test; p ≤ 0.05).

Parameters	Control	Mock-challenged				ECPs			
	0 h	3 h	6 h	24 h	48 h	3 h	6 h	24 h	48 h
**U SOD/mg prot**	22.18±1.59#	18.05±1.74**b**	27.30±0.67**a***	27.10±1.94	27.11±1.88	27.07±1.04**a***	19.50±1.69**b**	26.28±1.94	25.56±2.64

**CAT U/mg**	106.61±4.05#	107.28±4.94	98.70±3.07**b**	92.20±6.03*	92.86±5.89	108.99±3.58	110.32±3.59**a**	87.70±6.03*	97.62±4.55
**LPO (nmol/g wt)**	119.35±18.75	87.12±6.10**b**	90.45±4.14	94.27±6.42	101.07±5.65	131.94±18.24**a**	109.92±12.29	110.49±6.42	129.09±13.80
**GSH/GSSG ratio**	83.30±9.62#	151.78±19.90**a**	82.89±10.09	23.08±2.99*	32.53±6.57*	53.49±12.01**b***	95.44±27.20	33.15±2.99*	26.58±4.57*
**GSH**	2017.35±138.57#	2253.12±56.08**a**	1819.64±94.05	1611.51±112.88	2044.23±267.24	1963.25±124.28**b**	1524.29±121.53*	1414.77±112.88*	2419.85±169.55
**GSSG**	27.53±4.11#	24.18±9.38**b**	24.44±2.93	72.91±8.76*	72.27±10.56*	57.63±10.31**a***	34.40±12.79	72.36±8.76*	105.12±14.21*

Student's **t-test (*p*-values)**

Parameters	Mock-challenged x ECPs			
	**3 h**	**6 h**	**24 h**	**48 h**
**U SOD/mg prot**	<.001	<.001	0.735	0.638
**CAT U/mg**	0.781	0.024	0.528	0.529
**LPO (nmol/g wt)**	0.036	0.103	0.210	0.163
**GSH/GSSG ratio**	0.005	0.394	0.845	0.596
**GSH**	0.040	0.040	0.456	0.369
**GSSG**	0.003	0.565	0.618	0.126

Table S9: Oxidative stress biomarkers activity of the liver of European sea bass i.p. challenged with MB (Mock) or 5.5 x 10^5 CFU *T. maritimum* without ECPs (BWO) or 5.5 x 10^5 CFU *T. maritimum* with ECPs (BECPs). Data are expressed as mean ± SEM (n=12 *per* treatment). Different lowercase letters stand for significant differences in treatments among each time point, while different capital letters indicate differences in time among the same treatment (Two-Way ANOVA for interaction between factors, followed by Tukey's HSD or LSD for multiple comparisons, *p-value* ≤ 0.05). Different symbols represent significant differences between the control group (undisturbed) and the different treatment groups (*Student's* t-test; p ≤ 0.05).

Parameters	Control	Mock-challenged				BWO				BECPs			
	**0 h**	**3 h**	**6 h**	**24 h**	**48 h**	**3 h**	**6 h**	**24 h**	**48 h**	**3 h**	**6 h**	**24 h**	**48 h**
**U SOD/mg prot**	22.18±1.59	18.05±1.74	27.30±0.67	27.10±1.94	27.11±1.88	27.49±1.48	24.44±2.28	28.77±2.12	30.91±1.25	26.82±2.10	31.26±2.07	27.62±2.38	29.21±2.23
**CAT U/mg**	106.61±4.05	107.28±4.94	98.70±3.07	92.20±6.03	92.86±5.89	105.69±5.28	111.51±5.60	86.29±2.90	90.21±2.62	100.39±9.23	99.74±3.33	89.68±3.20	80.56±1.95
**LPO (nmol/g wt)**	119.35±18.75	87.12±6.10	90.45±4.14	94.27±6.42	101.07±5.65	104.48±11.37	96.98±6.02	123.86±12.99	121.19±17.61	120.60±12.56	111.68±12.38	139.66±15.84	145.45±10.02
**GSH/GSSG ratio**	83.30±9.62	151.78±19.90	82.89±10.09	23.08±2.99	32.53±6.57	113.23±37.90	103.06±25.61	17.87±7.08	28.29±3.80	97.87±35.53	39.88±8.51	14.82±4.55	33.42±12.09
**GSH**	2017.35±138.57	2253.12±56.08	1819.64±94.05	1611.51±112.88	2044.23±267.24	1915.60±126.41	1806.95±135.93	843.17±82.49	2303.20±154.43	1396.82±141.92	1489.66±114.11	816.78±75.68	2441.18±143.48
**GSSG**	27.53±4.11	24.18±9.38	24.44±2.93	72.91±8.76	72.27±10.56	30.85±6.04	31.74±6.54	60.28±6.28	90.58±12.03	25.84±5.65	59.06±13.41	87.68±15.13	107.60±13.68

One-Way ANOVA

Parameters	Time				Treatment		
	**3 h**	**6 h**	**24 h**	**48 h**	**Mock**	**BWO**	**BECPs**
**U SOD/mg prot**	-	-	-	-	-	-	-
**CAT U/mg**	A	A	B	B	-	-	-
**LPO (nmol/g wt)**	-	-	-	-	b	ab	a
**GSH/GSSG ratio**	A	A	B	C	b	ab	a
**GSH**	-	-	-	-	-	-	-
**GSSG**	A	A	B	B	-	-	-

2-Way ANOVA

Parameters	Time x Treatment											
	**Mock-challenged**				**BWO**				**BECPs**			
	**3 h**	**6 h**	**24 h**	**48 h**	**3 h**	**6 h**	**24 h**	**48 h**	**3 h**	**6 h**	**24 h**	**48 h**
**U SOD/mg prot**	Aa	Bab	B	B	Ab	Aa	AB	B	b	b	-	*
**CAT U/mg**	-	-	-	-	-	-	-	-	-	-	-	-
**LPO (nmol/g wt)**	-	-	-	-	-	-	-	-	-	-	-	-
**GSH/GSSG ratio**	-	-	-	-	-	-	-	-	-	-	-	-
**GSH**	ACa	BC	Ca	ABb	ABa	B	Cb	Aab	Ab	A	Bb	Ca
**GSSG**	-	-	-	-	-	-	-	-	-	-	-	-

2-Way ANOVA (*p*-values)

Parameters	**Time**	**Treatment**	**Time x Treatment**
**U SOD/mg prot**	0.014	0.015	0.025
**CAT U/mg**	<0.001	0.413	0.156
**LPO (nmol/g wt)**	0.090	<.001	0.751
**GSH/GSSG ratio**	<.001	<.001	0.240
**GSH**	<.001	<.001	<.001
**GSSG**	<.001	0.075	0.236

***Student's* t-test**

Parameters	Control	Mock-challenged				BWO				BECPs			
	**0 h**	**3 h**	**6 h**	**24 h**	**48 h**	**3 h**	**6 h**	**24 h**	**48 h**	**3 h**	**6 h**	**24 h**	**48 h**
**U SOD/mg prot**	#	-	*		*	*	-	*	-	-	*	-	*
**CAT U/mg**	#	-	-	*	-	-	-	*	*	-	-	*	*
**LPO (nmol/g wt)**	#	-	-		-	-	-	-	-	-	-	-	*
**GSH/GSSG ratio**	#	*	-	*	-	-	-	*	*	-	*	*	*
**GSH**	#	-	-	*	-	-	-	*	-	*	*	*	*
**GSSG**	#	-	-	*	-	-	-	*	*	-	*	*	*

Table S10: Quantitative expression of *il34*, *cxcr4*, *mmp9*, *mcsfr*, *mif*, *casp1*, *mhcII* and *hsp70* for head-kidney of European sea bass i.p. challenged with MB (Mock) or *T. maritimum*'s ECPs (ECPs). Data are expressed as mean ± SEM (n=9 *per* treatment). Different lowercase letters stand for significant differences between treatments among time points and different symbols represent significant differences between the control group (undisturbed) and the remaining groups (*Student's* t-test; $p \leq 0.05$).

Parameters	Control	Mock-challenged				ECPs			
	**0 h**	**3 h**	**6 h**	**24 h**	**48 h**	**3 h**	**6 h**	**24 h**	**48 h**
il34	1.05±0.12**#**	0.42±0.04*****	0.50±0.05*****	0.77±0.07**a**	1.17±0.06**a**	0.38±0.10*****	0.49±0.06*****	0.44±0.08**b***	0.53±0.03**b***
cxcr4	1.02±0.07**#**	1.17±0.07**a**	1.05±0.09**a**	1.07±0.10	1.05±0.10	0.78±0.05**b***	0.62±0.05**b***	0.91±0.07	0.85±0.04
mmp9	1.07±0.14**#**	3.33±0.99*****	5.32±0.58**a***	3.56±0.30**a***	2.55±0.25**a***	2.36±0.23*****	2.30±0.27**b***	1.48±0.38**b**	0.26±0.02**b***
mcsfr	1.01±0.06**#**	0.96±0.10	1.17±0.16	0.87±0.10	0.71±0.08**b***	0.88±0.07*****	1.38±0.08*****	0.97±0.08	1.02±0.09**a**
mif	1.09±0.17**#**	0.93±0.11	1.01±0.09	1.22±0.11**b**	0.79±0.08**b**	0.85±0.08	0.96±0.10	1.73±0.19**a***	1.99±0.21**a***
casp1	1.15±0.25	0.90±0.90	1.06±0.11	1.10±0.11	0.92±0.12	0.91±0.12	1.22±0.19	1.06±0.17	0.86±0.05
mhcII	1.03±0.09**#**	0.90±0.07	0.87±0.87	0.68±0.09*****	0.59±0.07*****	0.93±0.13	0.77±0.09*****	0.58±0.04*****	0.55±0.03*****
hsp70	1.01±0.06	1.06±0.09	1.08±0.06	1.02±0.08	1.01±0.10	0.92±0.07	1.10±0.08	1.21±0.12	1.14±0.11

Student's **t-test (*p*-values)**

Parameters	Mock-challenged x ECPs			
	**3 h**	**6 h**	**24 h**	**48 h**
il34	0.361	0.905	0.005	<.001
cxcr4	<.001	<.001	0.268	0.092
mmp9	0.439	<.001	<.001	<.001
mcsfr	0.587	0.144	0.311	0.028

mif	0.642	0.645	0.030	<.001
casp1	0.944	0.621	0.641	0.949
mhcII	0.944	0.760	0.430	0.948
hsp70	0.199	0.933	0.220	0.407

Table S11: Quantitative expression of *il34*, *cxcr4*, *mmp9*, *mcsfr*, *mif*, *casp1*, *mhcII* and *hsp70* for head-kidney of European sea bass i.p. challenged with MB (Mock) or 5.5 x 10^5 CFU *T. maritimum* without ECPs (BWO) or 5.5 x 10^5 CFU *T. maritimum* with ECPs (BECPs). Data are expressed as mean ± SEM (n=9 *per* treatment). Different lowercase letters stand for significant differences in treatments among each time point, while different capital letters indicate differences in time among the same treatment (Two-Way ANOVA for interaction between factors, followed by Tukey's HSD or LSD for multiple comparisons, *p-value* ≤ 0.05). Different symbols represent significant differences between the control group (undisturbed) and the different treatment groups (*Student's* t-test; p ≤ 0.05).

Parameters	**Control**	**Mock-challenged**				**BWO**				**BECPs**			
	**0 h**	**3 h**	**6 h**	**24 h**	**48 h**	**3 h**	**6 h**	**24 h**	**48 h**	**3 h**	**6 h**	**24 h**	**48 h**
il34	1.05±0.12	0.42±0.04	0.50±0.05	0.77±0.07	1.17±0.06	0.51±0.05	0.32±0.06	0.68±0.10	1.05±0.08	0.46±0.05	0.58±0.04	0.42±0.06	0.59±0.06
cxcr4	1.02±0.07	1.17±0.07	1.05±0.09	1.07±0.10	1.05±0.10	0.85±0.08	0.78±0.05	1.07±0.09	1.04±0.07	0.70±0.06	0.73±0.05	0.75±0.05	0.73±0.07
mmp9	1.07±0.14	3.33±0.99	5.32±0.58	3.56±0.30	2.55±0.25	4.79±0.72	5.80±0.57	2.24±0.19	1.39±0.11	2.37±0.32	2.20±0.30	0.93±0.15	0.87±0.13
mcsfr	1.01±0.06	0.96±0.10	1.17±0.16	0.87±0.10	0.71±0.08	0.96±0.09	1.30±0.09	0.80±0.12	0.87±0.07	1.15±0.08	1.49±0.13	0.74±0.05	0.90±0.09
mif	1.09±0.17	0.93±0.11	1.01±0.09	1.22±0.11	0.79±0.08	0.95±0.12	0.86±0.09	1.44±0.17	0.74±0.07	0.82±0.08	1.23±0.09	1.46±0.20	1.34±0.11
casp1	1.15±0.25	0.90±0.90	1.06±0.11	1.10±0.11	0.92±0.12	0.94±0.10	1.32±0.18	1.13±0.18	0.85±0.07	0.79±0.08	1.37±0.18	0.96±0.09	0.93±0.04
mhcII	1.03±0.09	0.90±0.07	0.87±0.87	0.68±0.09	0.59±0.07	1.10±0.13	1.03±0.15	0.69±0.09	0.73±0.09	0.84±0.08	0.64±0.08	0.53±0.02	0.61±0.04
hsp70	1.01±0.06	1.06±0.09	1.08±0.06	1.02±0.08	1.01±0.10	1.07±0.12	1.15±0.08	0.91±0.09	0.84±0.07	1.05±0.09	1.26±0.10	1.04±0.11	1.02±0.09

One-Way ANOVA

Parameters	**Time**				**Treatment**		
	**3 h**	**6 h**	**24 h**	**48 h**	**Mock**	**BWO**	**BECPs**
il34	-	-	-	-	-	-	-
cxcr4	-	-	-	-	b	b	a
mmp9	-	-	-	-	-	-	-
mcsfr	A	C	B	B	-	-	-
mif	-	-	-	-	-	-	-

casp1	B	A	AB	B	-	-	-
mhcII	B	AB	A	A	ab	b	a
hsp70	AB	A	AB	B	-	-	-

2-Way ANOVA

Parameters	**Time x Treatment**											
	**Mock-challenged**				**BWO**				**BECPs**			
	**3 h**	**6 h**	**24 h**	**48 h**	**3 h**	**6 h**	**24 h**	**48 h**	**3 h**	**6 h**	**24 h**	**48 h**
il34	A	Ab	aB	aC	A	aB	Aa	aC	AB	Ab	Bb	Ab
cxcr4	-	-	-	-	-	-	-	-	-	-	-	-
mmp9	Ab	aB	Ab	aA	aA	aA	aB	bC	Ab	Ab	Bc	Bc
mcsfr	-	-	-	-	-	-	-	-	-	-	-	-
mif	AB	ABab	A	bB	A	Ab	B	Ab	A	aB	B	aB
casp1	-	-	-	-	-	-	-	-	-	-	-	-
mhcII	-	-	-	-	-	-	-	-	-	-	-	-
hsp70	-	-	-	-	-	-	-	-	-	-	-	-

2-Way ANOVA (*p*-values)

Parameters	**Time**	**Treatment**	**Time x Treatment**
il34	<.001	0.002	<.001
cxcr4	0.344	<.001	0.202
mmp9	<.001	<.001	<.001
mcsfr	<.001	0.130	0.466
mif	<.001	0.023	0.016
casp1	0.002	0.857	0.633
mhcII	<.001	0.013	0.692
hsp70	0.021	0.271	0.775

***Student's* t-test**

Parameters	**Control**	**Mock-challenged**				**BWO**				**BECPs**			
	**0 h**	**3 h**	**6 h**	**24 h**	**48 h**	**3 h**	**6 h**	**24 h**	**48 h**	**3 h**	**6 h**	**24 h**	**48 h**

il34	#	*	*	-	-	*	*	*	-	*	*	*	*
cxcr4	#	-	-	-	-	-	*	-	-	*	*	*	*
mmp9	#	*	*	*	*	*	*	*	-	*	*	-	-
mcsfr	#	-	-	-	-	-	*	-	-	-	*	*	-
mif	#	-	-	-	-	-	-	-	-	-	-	-	-
casp1	#	-	-	-	-	-	-	-	-	-	-	-	-
mhcII	#	-	-	-	*	-	-	*	*	-	*	*	*
hsp70	-	-	-	-	-	-	-	-	-	-	-	-	-
